# Single-cell hemoprotein (heme-SCP) exerts the prebiotic potential to establish a healthy gut microbiota in small pet dogs

**DOI:** 10.1007/s10068-022-01195-9

**Published:** 2022-11-23

**Authors:** Seungki Lee, Ahyoung Choi, Kyung-Hoon Park, Seoyeon Lee, Hyunjin Yoon, Pil Kim

**Affiliations:** 1 Department of Biotechnology, The Catholic University of Korea, 43 Jibong-ro, Wonmi-gu, Bucheon, Gyeonggi 14662 South Korea; 2 HemoLab Ltd. Co., Bucheon, 14622 South Korea; 3 Department of Molecular Science and Technology, Ajou University, 206 World cup-ro, Yeongtong-gu, Suwon, Gyeonggi 16499 South Korea

**Keywords:** Single-cell hemoprotein, heme-SCP, Bacterial community, Dog, Prebiotics

## Abstract

To investigate the effect of the single-cell hemoprotein (heme-SCP) source on animals, a dog-treat (100 g for each dog) harboring 0.2% heme-SCP was manufactured and fed to seven pet dogs (< 10 kg) in a randomized manner (irrespective of owner’s feeding style, dogs’ health conditions, and staple diets), and the feces before and after the dog-treat diet were analyzed to define the structure of the microbiota. The total bacterial species of the seven dogs showed no difference (564–584), although the bacterial compositions varied significantly. The Firmicutes phylum increased (54.7–73.7%), showing differential species composition before and after heme-SCP intake. Proteobacteria, Bacteroidetes, and Fusobacteria decreased (5.4–3.8%, 32.9–16.8%, and 6.3–3.6%, respectively), which agreed with the previous observation of deliberate feeding. Therefore, it is conceivable that heme-SCP as a prebiotic can shape the gut microbiota regardless of the administration method.

**Supplementary Information:**

The online version contains supplementary material available at 10.1007/s10068-022-01195-9.

## Introduction

Humans coexist with trillions of microbes. Dysbiosis of the human microbiome is associated with numerous diseases, including inflammatory bowel disease, multiple sclerosis, diabetes (types I and II), allergies, asthma, autism, and even cancer (Turnbaugh et al., 2007; Ursell et al., 2012). With the development of next-generation sequencing (NGS) technology, the interactive relationship between human health and microbiome has become clearer (Jovel et al., 2016). The human gut microbiome consists of more than 400 bacterial species, most of which belong to only a few phyla, including Firmicutes, Bacteroidetes, Proteobacteria, and Actinobacteria (D’Argenio and Salvatore, 2015). Many bacterial species in the phylum Firmicutes have been reported to be closely related to human health; *Megamonas rupellensis* helps in type II diabetes treatment and glucose homeostasis (Díaz-Perdigones et al., 2022), *Faecalimonas umbilicata* contributes to human digestion by producing acetate (Sakamoto et al., 2018), and *Clostridium hiranonis* promotes secondary bile acid production by 7alpha-dehydroxylation activity (Kitahara et al., 2001). Species belonging to the Bacteroidetes phylum are also closely associated with human health, but their influence is disorienting amongst bacterial species or host health conditions. *Bacteroides vulgatus* is enriched in the human gut with intestinal diseases (e.g., Crohn’s disease) (Bloom et al., 2011; Dicksved et al., 2008), whereas it is depleted in patients suffering from coronary artery disease (Sieminska et al., 2021), and *Bacteroides plebeius*, frequently found in healthy individuals, is considered an indicator of healthy intestinal flora (Hernandez et al., 2022).

To unveil the multifarious roles of the microbiome, fecal microbiota are perpetually compared between healthy and diseased groups, including humans as well as animals representing a human-like microbiome. Although gut microbiome studies have been most extensively conducted in mice as an animal model, this species possesses physiological characteristics far different from humans, considering body size, metabolic rate, and life expectancy (Perlman, 2016). Emerging metagenomics data suggests that dogs represent gut microbiome closer to the human microbiome, compared with the microbiome of either mice or pigs (Coelho et al., 2018; Pallotti et al., 2022). Dogs became domesticated more than 14,000 years ago and are considered omnivorous, frequently sharing food resources with humans. This species often shows genetic disorders with similar pathophysiological and clinical features to the human counterpart (Pallotti et al., 2022). In this study, we investigated whether dietary intake of heme compounds could influence the composition and structure of the gut microbiota using a dog model.

Heme is an iron-bound biomolecule found in all organisms involved in respiratory metabolism, including animals, plants, and other microorganisms. Proteins harboring heme as a prosthetic group (hemoproteins or heme proteins) are widely distributed in nature and perform various biological functions; many globin proteins (i.e., hemoglobin, myoglobin, neuroglobin, leghemoglobin) attach or detach di-oxygen molecules, many cytochromes transport electrons in the respiratory chain or create radicals to degrade recalcitrant organics, heme-catalase and heme-peroxidase detoxify reactive oxygen species (ROS), diverse sensor hemoproteins deliver a variety of signals in or between cells. Many anaerobic bacteria, including lactic acid bacteria, are defective in heme production and utilization. Consequently, their respiratory metabolism is incomplete and less effective for energy generation (i.e., fermentation metabolism). Once the heme molecule is supplemented as a nutrient, many anaerobic bacteria produce more energy via respiration (Kim et al., 2021; Lechardeur et al., 2011). Likewise, numerous bacterial species colonizing the gut lack a complete heme biosynthesis pathway, but encode heme-requiring proteins (Gruss et al., 2012). Regarding the indispensable roles of heme in bacterial physiology, it is probable that dietary intake of heme compounds reshapes the composition and structure of the gut microbiota. We have previously observed that two female dogs with similar ages and body weights increased the abundance of the Firmicutes phylum and enhanced the gut microbiome diversity, when single-cell hemoprotein (equally, heme-SCP) was regularly administered for 6 days (Lee et al., 2021). In this study, to get an insight into the potential of heme-SCP as a prebiotic, ten pet dogs varying in age, body weight, sex, breed, and staple food were fed with heme-SCP and their gut microbiota structure were compared before and after the treatment.

## Materials and methods

### Manufacturing a dog-treat harboring single-cell hemoprotein (heme-SCP)

Heme-SCP ( 0.2 g, dried biomass of hemoprotein-rich bacterial cells; Cell Tech, Ltd. Co., Cheongju, Choongbuk, Korea) was mixed with acidified ingredients (frozen-dried pollack 20 g, sweet pumpkin 14.8 g, duck tenderloin 10 g, carrot 10 g, brown rice 5 g, salmon 5 g, sea mussel 5 g, cabbage 4 g, sweet potato 8 g, broccoli 3 g, coconut 3 g, tapioca starch 10 g, glycerin 5 g), dispersed in 10% vinegar solution, boiled for 1 h, extruded (2 cm diameter), cut to 1 cm thickness, dried at 62 °C for 5 h, and packed as 100 g units in a feed manufacturing facility (Hi-tech Korea Ltd. Co., Seoul, Korea) as a heme-SCP harboring dog-treat.

### Dog rearing and feces collection

Ten companion dogs (N = 10; 5 males and 5 females; age ranging from 6-month-old to 15-year-old; various breeds and lifestyles living in Korean households) were recruited, and the heme-SCP harboring dog-treat was provided to every dog owner. The dog-treat (100 g) was fed as a snack in an undesigned manner: different owner feeding styles, different dog lifestyles and health conditions, different breeds, no changes in the main diet, and no administration time limit. Fecal samples of dogs before and after the 100 g-dog-treat administration were collected in capped-tubes (50-mL tubes) and kept frozen until DNA extraction for bacterial taxonomic profiling. The owners were interviewed after the test to survey weights, preferences for the treat, and any abnormal behaviors or side effects in the dogs.

### Microbiome analysis

Frozen fecal samples were obtained from dog owners. Metagenomic DNA was extracted with FastDNA Spin kit (MP Biomedicals, Irvine, CA, USA) and the V3–V4 region of the bacterial 16S rRNA gene was PCR amplified using the barcoded universal primers (Yoon et al., 2017) of 341F (5′-AATGATACGGCGACCACCGAGATCTACAC-XXXXXXXX-TCGTCGGCAGCGTC-AGATGTGTATAAGAGACAG-CCTACGGGNGGCWGCAG-3′; underlining sequence indicates the target region primer-3′) and 805R (5′-CAAGCAGAAGACGGCATACGAGAT-XXXXXXXX-GTCTCGTGGGCTCGG-AGATGTGTATAAGAGACAG-GACTACHVGGGTATCTAATCC-3′). The amplifications were carried out under the following conditions: initial denaturation at 95 °C for 3 min, followed by 25 cycles of denaturation at 95 °C for 30 s, primer annealing at 55 °C for 30 s, and extension at 72 °C for 30 s, with a final elongation at 72 °C for 5 min. Purification of the amplicons was conducted using CleanPCR (CleanNA, Waddinxveen, Netherlands). The quality and product size were assessed on a Bioanalyzer 2100 (Agilent, Palo Alto, CA, USA) using a DNA 7500 chip. The pooled barcoded amplicons were sequenced using a MiSeq sequencer on the Illumina platform (CJ Bioscience, Inc., Seoul, Korea) according to the manufacturer’s specification. Taxonomic profiling of the microbiome was conducted using the EzBioCloud 16S rRNA database (Yoon et al., 2017). Statistical analysis was carried out using Mann–Whitney U-test (SPSS IBM, New York, NY, USA) to compare the variation in taxonomic profiles between samples. For analysis of alpha-diversity, the richness and diversity were determined by Shannon, Jackknife, and Simpson diversity indices using the in-house programs of CJ Bioscience, Inc. Sequencing coverage was calculated using Good’s method (Li et al., 2009).

### Bacterial growth curve

*Bacteroides vulgatus* ATCC8482 was cultivated in a mixed medium (BHI:MRS = 1:1) broth, and the optical density at 600 nm was measured to estimate bacterial growth. The broth medium was supplemented with heme-SCP (2 mg/mL) or hemin (Sigma-Aldrich, St. Louis, MO, USA) at 250 µM to examine the effect of heme on bacterial growth. BHI and MRS were purchased from BD Inc. (Sparks, MD, USA) and MBCell Inc. (Seoul, Korea), respectively.

## Results and discussion

Table 1 summarizes the information on the dogs (P1–P10) enrolled in this study. Many of the dogs were administered the treat for 2–3 weeks. However, P6 dog going through the toddler phase was fed exceptionally longer (30 days) according to the owner’s own volition, not spoiling a regular diet with staple food. No significant variations in weight or signs of possible illness were observed in any of the dogs. All owners reported that their dogs preferred the treat over their main diets. They also recognized the beneficial changes after administration, presumably attributable to the treat, including less diarrhea, better digestion, better stool consistency, and less putrid smelling stool, which are all associated with gut health. P4 (15-year-old) with a chronic digestive disorder was able to consume solid foods such as beef chunks and dried jerky during the treat test, although it returned to a poor meat-digestion state within a week after the test.

**Table 1:** Dogs included in this study

Code^a^	Dog name	Breed (gender)	Age (year)	Treat (100 g) consumption (days)	^2^Weight (Before)	Weight (after)^b^	Dogs’ preference (5-point scale)^c^	DNA extraction from fecal sample^d^	Owners’ comment^e^
P1	Choco	Poodle (F)	5	10	4.5	4.7	*****	F	Increased appetite
P2	Daebag	Shih Tzu (F)	6	15	4.8	4.7	*****	P	Less diarrhea
P3	Doongi	Speech (M)	6	14	4.8	4.7	*****	P	Increased appetite
P4	Puku	Poodle (M)	15	6	10.0	10.3	*****	P	Better meat digestion
P5	Jin-ju	Maltese (F)	13	22	3.4	3.6	*****	P	No recognizable difference
P6	Mini	Pomenarian (F)	0.5	30	2.3	2.3	****	P	Better stool consistency
P7	Schnauzer	Yorkshire Terrier (M)	6	9	5.4	5.7	*****	F	Better stool consistency
P8	Aaron	Chihuahua (F)	5	18	3.4	3.6	****	F	Less putrid smelling stool
P9	Arachi	Chihuahua (M)	7	20	3.3	3.3	*****	P	Less diarrhea
P10	Byeol-i	Schnauzer (M)	13	17	5.4	5.7	*****	P	Better meat digestion

^a^Ten dogs enrolled by the owners were provided with heme-SCP harboring dog-treat (100 g), and fecal samples before and after the dog treats were collected by the owners. Fecal samples from P1, P7, and P8, either before or after dog-treat, failed in 16S rRNA extraction because of late harvest or incorrect preservation, and the remainder from 7 dogs were analyzed in this study

^b^Body weights were measured by the owners using their own scales before and after the treat

^c^Dog’s preference for the treat was estimated based on owners’ judgement

^d^P/F: DNA extraction quality passed/failed

^e^Owner’s comments on the changes in their dogs’ health conditions during the test

Asterisks are not for statistical analysis. They indicate the dog preference toward the treat. Their preference was indicated using the number of asterisks from 1 to 5 as denoted in the table

**Fig. 1:**
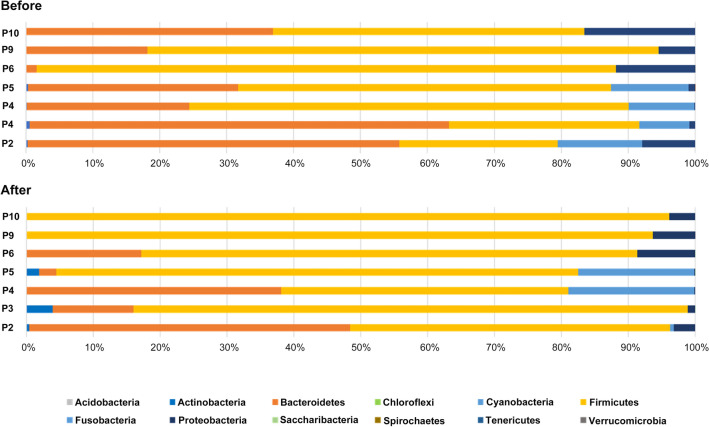
Alteration of fecal bacterial phyla of seven dogs before and after using the dog-treat. The fecal samples from seven dogs (P1 to P10 except P1, P7, and P8) before and after the dog treats were subjected to 16S rRNA sequencing, and bacterial abundance was analyzed at the phylum level. The alterations in species level are displayed in supplemental data (Table S1)

The fecal samples from P1, P7, and P8 dogs failed in the 16S rRNA sequencing because of DNA destruction either by delayed harvest or incorrect preservation. The microbiome profiles of the remaining 14 samples from seven dogs before and after the treat, are represented in Fig. 1 at the phylum level. The majority of the gut microbiota were from five phyla, namely: Actinobacteria, Bacteroidetes, Firmicutes, Fusobacteria, and Proteobacteria. However, the abundance of each phylum was markedly altered by the administration of dog-treat containing heme-SCP. For example, the gut microbiota of P5 exhibited a dramatic decrease in Bacteroidetes (31.4–2.6%) but significant increase in Firmicutes (55.7–78.0%) after the dog-treat consumption, even though the owner did not notice any changes in the dog’s health condition, such as ethological changes, altered bowel habit, and improvement of appetite. The dog-treat diet also led to directly opposing responses between Bacteroidetes (decreases) and Firmicutes (increases) in other entities, including P2, P3, P9, and P10. Notably, P9, suffering from frequent watery diarrhea before the test, did not have diarrhea during the intake of the dog-treat. Likewise, P3, whose microbiota was dominated by Firmicutes after the dog-treat consumption, was reported to smell less unpleasant. Intriguingly, the response of Bateroidetes and Firmicutes to the dog-treat was reversed in P4 (15-year-old) and P6 (0.5-year-old) dogs, showing increases in Bacteroidetes and decreases in Firmicutes. Considering that the rest are middle-aged, it is tempting to speculate that the potency of the dog-treat diet may vary in the infancy and old age groups, where the gut microbiome is immaturely established or vulnerable to exogenous stimuli. The profound effects of prebiotics on frailty and aging have been intensively explored in the recent studies (Jayanama and Theou, 2020; Mizukami et al., 2019). The effects of prebiotics on gut microbiota might be more noticeable in the senior dogs that suffer from health problems with aging, concomitantly experiencing microbial dysbiosis and metabolomic changes.

**Fig. 2:**
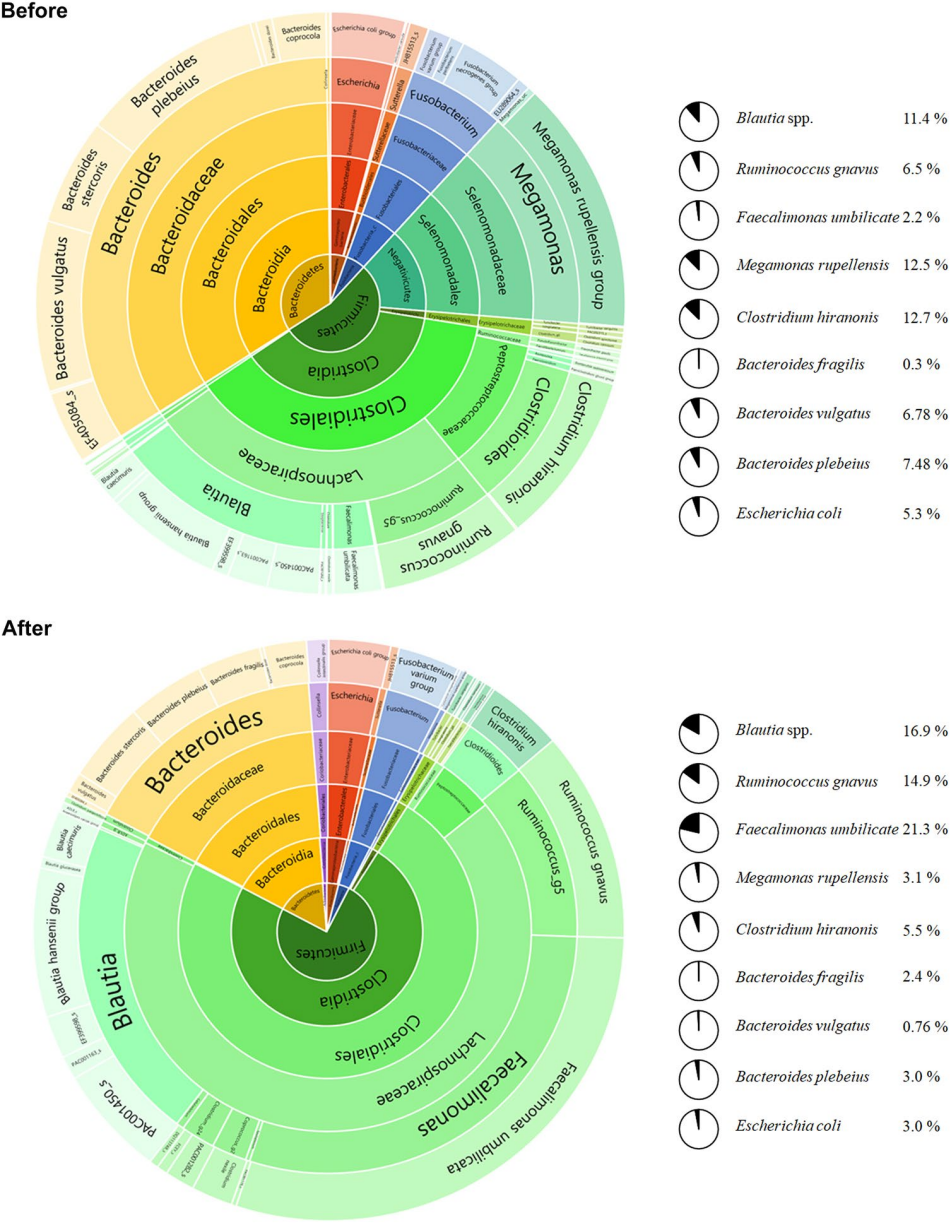
Average of seven dogs’ bacterial communities before and after the dog-treat. The microbiome data from seven dogs were integrated before and after the dog-treat diet and were averaged. Taxonomic profiles were compared before and after the dog-treat diet. The representative bacterial species showing significant changes due to the dog-treat diet are depicted in parallel

Because the dogs in this study were of different breeds, ages, health conditions, and were subjected to different feeding styles and main diets, many variables could influence microbiome conditions. To clarify the effect of randomized feeding of the heme-SCP-harboring treat, the microbiome data from seven dogs were integrated before and after the dog-treat diet and were averaged (Fig. 2). The total number of bacterial species identified using the EzBioCloud database after the treat was 584, which was comparable to the number before administration (564 species). The phylogenetic diversity (a measure of biodiversity that incorporates phylogenetic differences between species) (Faith and Baker, 2007) of seven dogs was also comparable (average phylogenetic diversity of 177.7–192.1). Again, the bacterial composition (in phylum units) was distinctly changed by treat supply. The proportion of Firmicutes increased (54.7–73.7%), whereas that of Bacteroides decreased (32.9–16.8%) after the test.

When the Firmicutes phylum was dissected at the species level, the dog-treat diet increased the abundance of *Blautia* spp. (14-fold, 1.4–16.9%), *Ruminococcus gnavus* (2.3-fold, 6.5–14.9%), and *Faecalimonas umbilicate* (9.7-fold, 2.2–21.3%) and decreased the abundance of *M. rupellensis* (0.24-fold, 12.5–3.1%) and *C. hiranonis* (0.42-fold, 12.7–5.5%) (Fig. 2). *Blautia* spp. have been reported to flourish in low visceral (hidden) fat humans (Ozato et al., 2019), thrive in humans with whole grain-induced immunological improvements (Martínez et al., 2013), but perish in dogs with acute hemorrhagic diarrhea syndrome (AHDS) (Guard et al., 2015). *Ruminococcus gnavus* is enriched in the infant human gut and has been suggested as a host immune educator (Chua et al., 2018; Sagheddu et al., 2016). *F. umbilicate*, an acetate-producing bacterial species, contributes to the establishment of gut microbial flora by enriching acetate-metabolizing butyrate-producing bacteria (Duncan et al., 2004; Sakamoto et al., 2017). *M. rupellensis*, an aerobe that produces short-chain fatty acids in the gut, was reported to shrink in the host with reduced glucose metabolism (Martín-Núñez et al., 2019). *C. hiranonis* has been reported to metabolize primary bile acids to secondary bile acids (Ridlon et al., 2020), some (i.e., deoxycholic acid) of which may trigger cancer in the intestines of many animals (Pai et al., 2004; Yoshimoto et al., 2013).

The intake of dog-treat reduced the proportion of Bacteroidetes phylum per se, but at the level of bacterial species, its influence was differential. The dog-treat diet decreased the abundance of *B. vulgatus* (0.11-fold, 6.78–0.76%) and *B. plebeius* (0.4-fold, 7.48–3%), whereas it increased the abundance of *Bacteroides fragilis* (8.3-fold, 0.3–2.4%) (Fig. 2). In general, the Bacteroidetes phylum is regarded as a commensal bacterium in the healthy gut, but the likelihood of pathogenicity varies depending on the bacterial species and host health conditions. For example, *Bacteroides fragilis* is the primary species causing *Bacteroides* infection when displaced into the bloodstream (Tajkarimi and Wexler, 2017). *B. plebeius* in the gut of people with Japanese descent is positively linked to the host’s complex carbohydrate (found in red seaweed) degradation and energy metabolism (Hehemann et al., 2012) but is also enriched in patients with cardiovascular disease (Liu et al., 2019). *B. vulgatus* has been reported to flourish in the gut of patients with intestinal disease (Crohn’s disease) (Dicksved et al., 2008), and decline in the gut of patients with coronary artery disease (Sieminska et al., 2021).

**Fig. 3:**
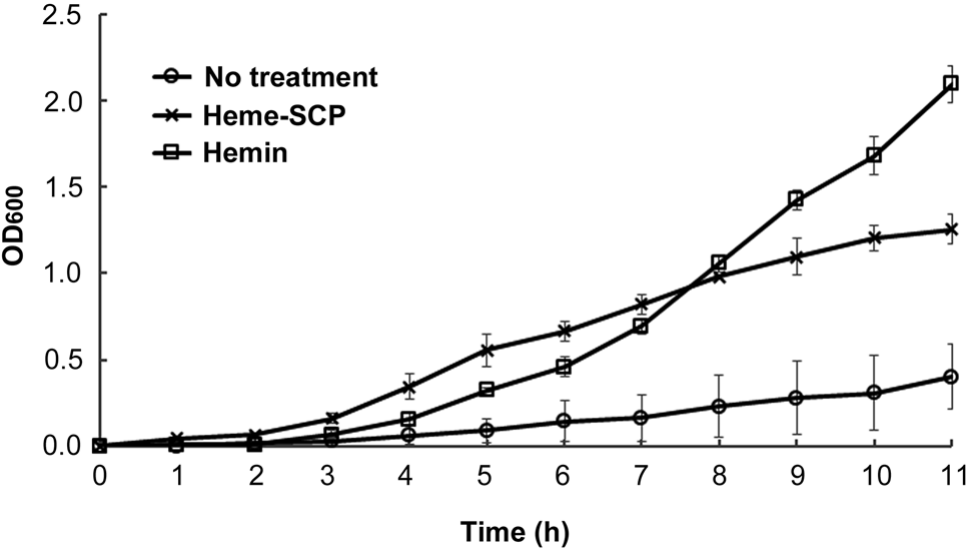
Effect of heme-SCP on the growth of *B. vulgatus.* *B. vulgatus* was cultivated in the presence of heme-SCP (2 mg/mL) or hemin (250 µM). The absorbance at 600 nm was measured every hour for 11 h and the values from three independent tests were plotted

To assess whether the altered bacterial composition was attributable to the iron compounds added to the dog-treat, the growth of *B. vulgatus* was compared in the presence and absence of heme-SCP. Interestingly, the addition of heme-SCP increased the bacterial growth rate, which was in contrast to the metagenome profiles in vivo (Fig. 3). The positive role of heme in *B. vulgatus* growth was validated by the addition of hemin, a ferric iron-containing porphyrin compound. The bacterial requirement for iron differs between bacterial species. To date, the detailed mechanism of iron acquisition in *B. vulgatus* has not been identified. However, Sieminska et al. (2021) recently claimed that *B. vulgatus* exploited a Bvu-based hemophore system to scavenge heme compounds, thereby promoting bacterial growth and virulence in the presence of heme (Sieminska et al., 2021). The discrepancy between the in vitro and in vivo analyses might be due to the differences in environment. *Bacteroides* spp. grow much slower than other commensal bacterial species, especially those belonging to the *Enterobacteriaceae* family, including harmless symbionts and opportunistic pathogens. Enteric bacteria such as *Escherichia coli* possess a variety of iron-sequestration strategies, including siderophores and iron transporters (Sousa Geros et al., 2020). Therefore, it is likely that other bacterial species outcompete *B. vulgatus* for scavenging heme and other iron equivalents in the gut environment.

The dog-treat diet marginally decreased the abundance of the Proteobacteria phylum from 5.4 to 3.8%, and the dominant bacterial species was *E. coli* (decreased from 5.3 to 3.0%), the most common human gut microorganism known as a fast monosaccharide degrader (Fig. 2). In the context of gut health, *E. coli* species exhibits multifaceted roles among bacterial strains. Although many *E. coli* strains are commensal, some strains are pathogenic and cause diseases in cases of microbiome perturbations, and some (e.g. *E. coli* strain Nissle 1917) are probiotics that decelerate the occurrence of intestinal inflammation and diseases (Gronbach et al., 2010).

Altogether, the undesigned feeding of heme-SCP-harboring treat, regardless of feeding style, lifestyle, health condition, dog breed, and main diet, reshaped the structure of the gut microbiome, showing a tendency to improve gut health: more fat degradation (*Blautia* spp. up), more immune lesson (*Ruminococcus gnavus* up), more diversity by enriching butyrate-producing beneficial bacteria (*F. umbilicate* up), less chance of carbohydrate digestion (*M. rupellensis* down, *B. plebeius* down, *E. coli* down), and a lower chance of intestinal diseases (*C. hiranonis* down, *B. vulgatus* down). These results are in accordance with previous observations where the controlled feeding of heme-SCP enriched Firmicutes in a dog model, and the heme-SCP addition benefited the growth of *Lactobacillus gasseri*, a representative of Firmicutes (Lee et al., 2021).

## Supplementary Information

Below is the link to the electronic supplementary material.

Supplementary material 1 (DOCX 80.8 kb)
