# Supplementary material for: Single-cell hemoprotein (heme-SCP) exerts the prebiotic potential to establish a healthy gut microbiota in small pet dogs
Source: Food Sci Biotechnol. 2022 Nov 23;32(4):489–96. doi: 10.1007/s10068-022-01195-9 (PMC9992493; doi:10.1007/s10068-022-01195-9)
Supplement: Supplementary file 1 — Supplementary material 1 (DOCX 80.8 kb) [file 10068_2022_1195_MOESM1_ESM.docx]

Table S1. **Alteration of fecal bacterial species of seven dogs before and after using the dog-treat**

|  | P2-B | P3-B | P4-B | P5-B | P6-B | P9-B | P10-B | Average ± SD |  | P2-A | P3-A | P4-A | P5-A | P6-A | P9-A | P10-A | Average ± SD | P value |
| --- | --- | --- | --- | --- | --- | --- | --- | --- | --- | --- | --- | --- | --- | --- | --- | --- | --- | --- |
| *Diversity index (Shannon) | 2.74 | 2.33 | 1.79 | 1.92 | 2.199 | 1.244 | 2.00 | 2.0318571  ±0.4674457 |  | 2.5 | 2.19 | 1.93 | 2.31 | 2.23 | 0.6 | 1.92 | 1.954285 0.6313704 | 0.5495126 |
| **Diversity index (Jackknife) | 116 | 99 | 96 | 96 | 230 | 288 | 142 | 152.42857  ±76.437027 |  | 108 | 157.9 | 112 | 136 | 139.6 | 137 | 169.6 | 137.1571 22.260418 | 0.6181854 |
| ***Diversity index (Simpson) | 0.093 | 0.152 | 0.244 | 0.219 | 0.162 | 0.48 | 0.213 | 0.223285  ±0.1240077 |  | 0.125 | 0.179 | 0.196 | 0.135 | 0.141 | 0.756 | 0.255 | 0.255285 0.2252974 | 0.4970096 |
| ****Good's coverage (%) | 99.9 | 99.9 | 99.9 | 99.9 | 99.8 | 99.9 | 99.9 | 99.88571  ±0.0377964 |  | 99.9 | 99.9 | 100 | 99.9 | 99.9 | 99.9 | 99.9 | 99.91428 0.0377964 | 0.1723083 |
| AB255102_s | 0 | 0 | 0 | 0 | 0.0031 | 0 | 0 | 0.000443  ±0.0011717 |  | 0 | 0 | 0 | 0 | 0 | 0 | 0 | 0±0 | 0.3559177 |
| AB606257_s | 0 | 0 | 0 | 0 | 0 | 0 | 0 | 0±0 |  | 0 | 0 | 0 | 0 | 0 | 0 | 0.003 | 0.000428 0.0011339 | 0.3559177 |
| AB606281_s | 0 | 0.0032 | 0 | 0 | 0 | 0 | 0 | 0.000457±0.0012095 |  | 0 | 0 | 0.0026 | 0 | 0 | 0 | 0.0059 | 0.001214 0.0022821 | 0.5044977 |
| AB626913_s | 0 | 0 | 0 | 0 | 0 | 0 | 0 | 0±0 |  | 0 | 0 | 0 | 0 | 0.0039 | 0 | 0 | 0.000557 0.0014741 | 0.3559177 |
| AB626927_s | 0 | 0 | 0 | 0 | 0.0031 | 0 | 0.0033 | 0.0009143  ±0.0015625 |  | 0 | 0 | 0 | 0 | 0.0039 | 0.0028 | 0 | 0.000957 0.0016652 | 0.9517145 |
| ADLB_s | 1.0379 | 0.1735 | 0 | 0 | 0.1661 | 0 | 0.2671 | 0.2349429  ±0.3696582 |  | 0.2308 | 1.3074 | 0 | 0 | 0.4936 | 0.0028 | 0.4834 | 0.359714 0.4715448 | 0.586613 |
| AM930332_s | 0 | 0 | 0 | 0 | 0.0031 | 0 | 0 | 0.0004429  ±0.0011717 |  | 0 | 0 | 0 | 0 | 0 | 0 | 0 | 0±0 | 0.3559177 |
| AP012202_s group | 0.0028 | 0 | 0 | 0 | 0 | 0 | 0 | 0.0004  ±0.0010583 |  | 0 | 0 | 0 | 0 | 0 | 0 | 0 | 0±0 | 0.3559177 |
| AP017369_s | 0 | 0 | 0 | 0 | 0.0063 | 0 | 0 | 0.0009  ±0.0023812 |  | 0 | 0 | 0 | 0 | 0 | 0 | 0 | 0±0 | 0.3559177 |
| AF349416_s | 0 | 0 | 0 | 0 | 0 | 0 | 0 | 0±0 |  | 0 | 0 | 0.0026 | 0 | 0 | 0 | 0 | 0.000371±0.0009827 | 0.3559177 |
| AJ315979_s | 0 | 0 | 0 | 0 | 0 | 0 | 0 | 0±0 |  | 0 | 0 | 0 | 0.0079 | 0 | 0 | 0 | 0.001128±0.0029859 | 0.3559177 |
| AM500787_s | 0 | 0 | 0 | 0 | 0 | 0 | 0 | 0±0 |  | 0 | 0 | 0 | 0 | 0 | 0.0028 | 0 | 0.0004 ±0.0010583 | 0.3559177 |
| AY375144_s | 0 | 0 | 0 | 0 | 0 | 0 | 0 | 0±0 |  | 0 | 0 | 0 | 0 | 0.0039 | 0 | 0 | 0.0005571 ±0.0014741 | 0.3559177 |
| AY957891_g_uc | 0 | 0 | 0 | 0 | 0 | 0 | 0 | 0±0 |  | 0 | 0 | 0 | 0 | 0 | 0 | 0.0059 | 0.0008429 ±0.00223 | 0.3559177 |
| Acidovorax delafieldii group | 0 | 0 | 0 | 0 | 0 | 0 | 0 | 0±0 |  | 0.0055 | 0 | 0 | 0 | 0 | 0 | 0 | 0.0007857 ±0.0020788 | 0.3559177 |
| Acidovorax_uc | 0 | 0 | 0 | 0 | 0 | 0 | 0 | 0±0 |  | 0.0027 | 0 | 0 | 0 | 0 | 0 | 0 | 0.0003857 ±0.0010205 | 0.3559177 |
| Acinetobacter pittii group | 0 | 0 | 0 | 0 | 0 | 0 | 0 | 0±0 |  | 0 | 0.003 | 0 | 0 | 0 | 0 | 0 | 0.0004286 ±0.0011339 | 0.3559177 |
| Acinetobacter proteolyticus group | 0 | 0 | 0 | 0.0024 | 0 | 0 | 0 | 0.0003429  ±0.0009071 |  | 0 | 0 | 0 | 0 | 0 | 0 | 0 | 0±0 | 0.3559177 |
| Actinomyces bowdenii | 0 | 0.0032 | 0 | 0 | 0 | 0 | 0 | 0.0004571  ±0.0012095 |  | 0 | 0.009 | 0 | 0.0026 | 0 | 0 | 0 | 0.0016571 ±0.0033798 | 0.207554 |
| Actinomyces canis | 0 | 0 | 0 | 0.0071 | 0.0031 | 0 | 0 | 0.0014571  ±0.0027434 |  | 0 | 0 | 0 | 0.103 | 0 | 0 | 0 | 0.0147143 ±0.0389303 | 0.3731971 |
| Actinomyces coleocanis | 0 | 0 | 0.0099 | 0.0024 | 0 | 0 | 0 | 0.0017571  ±0.0037004 |  | 0 | 0.003 | 0 | 0.0106 | 0 | 0 | 0 | 0.0019429 ±0.0039778 | 0.9302719 |
| Actinomyces hordeovulneris | 0 | 0 | 0 | 0 | 0 | 0 | 0 | 0±0 |  | 0 | 0 | 0 | 0 | 0 | 0.0055 | 0 | 0.0007857 ±0.0020788 | 0.3559177 |
| Actinomyces naeslundii | 0 | 0 | 0 | 0 | 0.0031 | 0 | 0 | 0.0004429  ±0.0011717 |  | 0 | 0 | 0 | 0.0026 | 0 | 0 | 0 | 0.0003714 ±0.0009827 | 0.9125487 |
| Actinomyces viscosus group | 0 | 0 | 0 | 0 | 0 | 0 | 0 | 0±0 |  | 0 | 0 | 0 | 0.0026 | 0 | 0 | 0 | 0.0003714 ±0.0009827 | 0.3559177 |
| Actinomyces_uc | 0 | 0 | 0 | 0 | 0 | 0 | 0 | 0±0 |  | 0 | 0 | 0 | 0.0106 | 0 | 0 | 0 | 0.0015143 ±0.0040064 | 0.3559177 |
| Actinosynnema pretiosum group | 0 | 0 | 0 | 0 | 0 | 0 | 0 | 0±0 |  | 0 | 0.003 | 0 | 0 | 0 | 0 | 0 | 0.0004286 ±0.0011339 | 0.3559177 |
| Agathobacter rectalis | 0 | 0 | 0 | 0.0024 | 0 | 0.0059 | 0 | 0.0011857  ±0.0022631 |  | 0 | 0 | 0 | 0 | 0.0039 | 0 | 0 | 0.0005571 ±0.0014741 | 0.5960866 |
| Agathobacter ruminis | 0 | 0 | 0 | 0 | 0 | 0 | 0 | 0±0 |  | 0 | 0 | 0 | 0 | 0 | 0 | 0.0059 | 0.0008429 ±0.00223 | 0.3559177 |
| Akkermansia muciniphila | 0 | 0 | 0 | 0 | 0.0031 | 0 | 0 | 0.0004429  ±0.0011717 |  | 0 | 0 | 0 | 0 | 0 | 0.0028 | 0 | 0.0004 ±0.0010583 | 0.9491299 |
| Anaerobiospirillum succiniciproducens | 0 | 0 | 0 | 0 | 0 | 0 | 0 | 0±0 |  | 0 | 0 | 0 | 0.0555 | 0 | 0 | 0 | 0.0079286 ±0.020977 | 0.3559177 |
| Anaerostipes caccae group | 0.1273 | 0.0726 | 0 | 0 | 0.3854 | 0 | 0.1936 | 0.1112714  ±0.1419042 |  | 0.3874 | 0.075 | 0 | 0.0528 | 0.2198 | 0 | 0.0208 | 0.1079714 ±0.1447017 | 0.9542125 |
| Anaerostipes hadrus group | 0.0028 | 0.0032 | 0.0049 | 0.0047 | 0 | 0.003 | 0 | 0.0026571  ±0.0019899 |  | 0 | 0 | 0 | 0 | 0.0039 | 0.0028 | 0.003 | 0.0013857 ±0.0017611 | 0.3842997 |
| Anaerotruncus colihominis | 0 | 0 | 0 | 0 | 0 | 0 | 0 | 0±0 |  | 0 | 0.003 | 0 | 0 | 0 | 0 | 0 | 0.0004286 ±0.0011339 | 0.3559177 |
| Aneurinibacillus thermoaerophilus | 0 | 0 | 0 | 0 | 0 | 0 | 0 | 0±0 |  | 0 | 0 | 0 | 0 | 0.0231 | 0 | 0 | 0.0033 ±0.008731 | 0.3559177 |
| Aneurinibacillus thermoaerophilus | 0 | 0 | 0 | 0 | 0.0251 | 0 | 0 | 0.0035857  ±0.0094869 |  | 0 | 0 | 0 | 0 | 0 | 0 | 0 | 0±0 | 0.3559177 |
| Arcanobacterium_uc | 0.0028 | 0 | 0 | 0 | 0 | 0 | 0 | 0.0004  ±0.0010583 |  | 0 | 0 | 0 | 0 | 0 | 0 | 0 | 0±0 | 0.3559177 |
| Arthrobacter globiformis group | 0 | 0 | 0 | 0.0024 | 0 | 0 | 0 | 0.0003429  ±0.0009071 |  | 0 | 0.003 | 0 | 0.0026 | 0 | 0 | 0 | 0.0008 ±0.0013711 | 0.3231644 |
| Bacillus clausii group | 0 | 0 | 0 | 0 | 0.0031 | 0 | 0 | 0.0004429  ±0.0011717 |  | 0 | 0 | 0 | 0 | 0 | 0.1718 | 0.0653 | 0.0338714 ±0.0655088 | 0.2274107 |
| Bacillus coagulans group | 0 | 0 | 0 | 0 | 0.0031 | 0 | 0 | 0.0004429  ±0.0011717 |  | 0.0027 | 0 | 0 | 0 | 0 | 0 | 0 | 0.0003857 ±0.0010205 | 0.9311067 |
| Bacillus dakarensis | 0 | 0 | 0 | 0 | 0 | 0 | 0 | 0±0 |  | 0.0027 | 0 | 0 | 0 | 0 | 0 | 0 | 0.0003857 ±0.0010205 | 0.3559177 |
| Bacillus fumarioli group | 0 | 0 | 0 | 0 | 0 | 0 | 0 | 0±0 |  | 0 | 0.003 | 0 | 0 | 0 | 0 | 0 | 0.0004286 ±0.0011339 | 0.3559177 |
| Bacillus halosaccharovorans | 0 | 0 | 0 | 0 | 0 | 0 | 0 | 0±0 |  | 0.0055 | 0 | 0 | 0 | 0 | 0 | 0 | 0.0007857 ±0.0020788 | 0.3559177 |
| Bacillus kochii | 0 | 0 | 0 | 0 | 0.0031 | 0 | 0 | 0.0004429  ±0.0011717 |  | 0 | 0 | 0 | 0 | 0.0039 | 0 | 0 | 0.0005571 ±0.0014741 | 0.3559177 |
| Bacillus licheniformis group | 0 | 0 | 0 | 0 | 0.0219 | 0 | 0 | 0.0031286  ±0.0082774 |  | 0 | 0 | 0 | 0 | 0.0077 | 0 | 0 | 0.0011 ±0.0029103 | 0.3559177 |
| Bacillus marisflavi group | 0 | 0 | 0 | 0 | 0 | 0 | 0 | 0±0 |  | 0 | 0 | 0 | 0 | 0.0077 | 0 | 0 | 0.0011 ±0.0029103 | 0.3559177 |
| Bacillus oleronius group | 0 | 0 | 0 | 0 | 0 | 0 | 0 | 0±0 |  | 0 | 0 | 0.0026 | 0 | 0 | 0 | 0 | 0.0003714 ±0.0009827 | 0.3559177 |
| Bacillus pumilus group | 0 | 0 | 0 | 0 | 0.0063 | 0 | 0 | 0.0009  ±0.0023812 |  | 0 | 0 | 0 | 0 | 0.0077 | 0 | 0 | 0.0011 ±0.0029103 | 0.3559177 |
| Bacillus thermolactis group | 0 | 0 | 0 | 0 | 0.0094 | 0 | 0 | 0.0013429  ±0.0035529 |  | 0 | 0 | 0 | 0 | 0 | 0 | 0 | 0±0 | 0.3559177 |
| Bacteroides coprocola | 8.8516 | 0.0063 | 3.9807 | 5.9201 | 0 | 0 | 0 | 2.6798143  ±3.6280065 |  | 0 | 0 | 0 | 0 | 0 | 0 | 0 | 0±0 | 0.0984736 |
| Bacillus smithii | 0 | 0 | 0 | 0 | 0 | 0 | 0 | 0±0 |  | 0 | 0 | 0 | 0 | 0.0077 | 0 | 0 | 0.0011 ±0.0029103 | 0.3559177 |
| Bacillus subtilis group | 0 | 0 | 0 | 0 | 0 | 0 | 0 | 0±0 |  | 0 | 0 | 0 | 0 | 0.0193 | 0 | 0 | 0.0027571 ±0.0072947 | 0.3559177 |
| Bacteroides caccae | 0 | 0 | 0 | 0 | 0 | 0 | 0 | 0±0 |  | 0 | 0 | 0 | 0 | 0 | 0 | 0.003 | 0.0004286 ±0.0011339 | 0.3559177 |
| Bacteroides coprocola | 0 | 0 | 0 | 0 | 0 | 0 | 0 | 0±0 |  | 10.7881 | 0.012 | 6.072 | 0.729 | 0 | 0 | 0 | 2.5144429 ±4.2725268 | 0.1704625 |
| Bacteroides dorei | 4.4088 | 0.0063 | 0 | 0 | 0 | 0.0059 | 0 | 0.6315714  ±1.6656037 |  | 1.0167 | 0.003 | 0 | 0 | 0 | 0 | 0 | 0.1456714 ±0.3840891 | 0.3545079 |
| Bacteroides faecis | 0 | 0 | 0 | 0 | 0.0063 | 0 | 0 | 0.0009  ±0.0023812 |  | 0 | 0 | 0 | 0 | 0 | 0 | 0 | 0±0 | 0.3559177 |
| Bacteroides fragilis | 0.0085 | 0.0063 | 0 | 0.0047 | 1.5416 | 0.0059 | 0 | 0.2238571  ±0.5810788 |  | 0.0027 | 0 | 0 | 0 | 17.0883 | 0.0028 | 0 | 2.4419714 ±6.458424 | 0.3565845 |
| Bacteroides ovatus group | 0 | 0.0063 | 0 | 0 | 0.0031 | 0.003 | 0 | 0.0017714  ±0.0024608 |  | 0.0027 | 0 | 0 | 0 | 0 | 0 | 0 | 0.0003857 ±0.0010205 | 0.2600346 |
| Bacteroides plebeius | 17.5165 | 27.6607 | 1.747 | 5.4151 | 0 | 0.003 | 0 | 7.4774714  ±10.90053 |  | 20.1473 | 0.4588 | 0.0157 | 0.3486 | 0.0039 | 0 | 0 | 2.9963286 ±7.5652725 | 0.2929593 |
| Bacteroides sartorii | 0 | 0 | 0 | 0 | 0 | 0 | 0 | 0±0 |  | 0 | 0 | 0 | 0 | 0 | 0 | 0.003 | 0.0004286 ±0.0011339 | 0.3559177 |
| Bacteroides salyersiae | 0 | 0 | 0 | 0 | 0 | 0.003 | 0 | 0.0004286  ±0.0011339 |  | 0 | 0 | 0 | 0 | 0 | 0 | 0 | 0±0 | 0.3559177 |
| Bacteroides sartorii | 0 | 0 | 0 | 0 | 0 | 0.003 | 0 | 0.0004286  ±0.0011339 |  | 0 | 0 | 0 | 0 | 0 | 0 | 0 | 0±0 | 0.3559177 |
| Bacteroides stercoris | 11.4109 | 18.1818 | 0.0049 | 0.0095 | 0 | 0 | 0 | 4.2295857  ±7.4784227 |  | 12.9836 | 9.107 | 0.0052 | 0.0079 | 0 | 0 | 0 | 3.1576714 ±5.5032594 | 0.4580971 |
| Bacteroides uniformis | 0 | 0 | 0.0025 | 0 | 0 | 0 | 0 | 0.0003571  ±0.0009449 |  | 0 | 0.003 | 0 | 0 | 0.0039 | 0 | 0 | 0.0009857 ±0.0017034 | 0.4682794 |
| Bacteroides vulgatus | 12.904 | 16.5952 | 0.0049 | 0.0047 | 0.0031 | 17.9187 | 0.0167 | 6.7781857  ±8.576979 |  | 2.9897 | 2.4889 | 0.0026 | 0.0053 | 0.0154 | 0.0277 | 0.0089 | 0.7912143 ±1.3386503 | 0.089054 |
| Bacteroides xylanisolvens group | 0 | 0 | 0 | 0 | 0.0094 | 0.0148 | 36.9099 | 5.2763  ±13.949108 |  | 0 | 0 | 0 | 0 | 0.0193 | 0 | 0 | 0.0027571 ±0.0072947 | 0.3558486 |
| Bacteroides_uc | 0.3196 | 0.1577 | 0.0025 | 0.0024 | 0 | 0.1534 | 0.01 | 0.0922286  ±0.1232303 |  | 0.0659 | 0.009 | 0.0105 | 0 | 0.0077 | 0 | 0 | 0.0133 ±0.0236471 | 0.0948046 |
| Bifidobacterium adolescentis group | 0.0028 | 0 | 0 | 0 | 0 | 0.003 | 0 | 0.0008286  ±0.0014162 |  | 0.0027 | 0.009 | 0.0026 | 0 | 0 | 0 | 0 | 0.0020429 ±0.0033125 | 0.4297972 |
| Bifidobacterium animalis group | 0 | 0 | 0 | 0 | 0 | 0.003 | 0 | 0.0004286  ±0.0011339 |  | 0 | 0 | 0 | 0 | 0 | 0 | 0 | 0±0 | 0.3559177 |
| Bifidobacterium catenulatum group | 0 | 0 | 0 | 0.0024 | 0 | 0.0089 | 0.0033 | 0.0020857  ±0.0033017 |  | 0.0055 | 0 | 0 | 0 | 0 | 0.0028 | 0.003 | 0.0016143 ±0.0021927 | 0.7307001 |
| Bifidobacterium longum group | 0 | 0 | 0 | 0 | 0 | 0.003 | 0 | 0.0004286  ±0.0011339 |  | 0 | 0 | 0 | 0 | 0 | 0 | 0 | 0±0 | 0.3559177 |
| Bifidobacterium pseudolongum group | 0 | 0 | 0 | 0 | 0 | 0 | 0.0033 | 0.0004714  ±0.0012473 |  | 0 | 0 | 0 | 0 | 0 | 0 | 0.003 | 0.0004286 ±0.0011339 | 0.3559177 |
| Blautia caecimuris | 0.4892 | 6.968 | 0.0272 | 0.0047 | 0 | 0 | 0.02 | 1.0727286  ±2.6057077 |  | 1.5553 | 11.458 | 0.0052 | 0.0053 | 0 | 0 | 0.083 | 1.8724 ±4.2655694 | 0.2532964 |
| Blautia coccoides | 0 | 0 | 0 | 0 | 0.0501 | 0 | 0.0267 | 0.0109714  ±0.0199177 |  | 0 | 0 | 0 | 0.0053 | 0.0077 | 0 | 0.0475 | 0.0086429 ±0.01742 | 0.7596399 |
| Blautia faecis | 0 | 0 | 0 | 0.0047 | 0 | 0 | 0 | 0.0006714  ±0.0017764 |  | 0 | 0.003 | 0 | 0 | 0 | 0 | 0 | 0.0004286 ±0.0011339 | 0.785837 |
| Blautia glucerasea | 0.3676 | 1.1955 | 0.0618 | 0 | 0 | 0 | 0.0701 | 0.2421429  ±0.440199 |  | 0.8985 | 2.198 | 0 | 0 | 0.0039 | 0 | 0 | 0.4429143 ±0.84314 | 0.2437286 |
| Blautia hansenii group | 0.806 | 3.0124 | 2.2733 | 0.0024 | 27.6704 | 0.0148 | 20.2177 | 7.7138571  ±11.348815 |  | 2.1791 | 5.4126 | 1.4513 | 20.2234 | 12.931 | 0.0166 | 22.5501 | 9.2520143 ±9.3243102 | 0.7035203 |
| Blautia luti | 0.0028 | 0 | 0 | 0 | 0 | 0 | 0.0033 | 0.0008714  ±0.0014952 |  | 0.0027 | 0.003 | 0 | 0 | 0 | 0 | 0 | 0.0008143 ±0.0013934 | 0.936503 |
| Blautia obeum | 0 | 0 | 0 | 0.0024 | 0.0031 | 0 | 0 | 0.0007857  ±0.001357 |  | 0 | 0 | 0 | 0 | 0 | 0.0028 | 0 | 0.0004 ±0.0010583 | 0.6145489 |
| Blautia schinkii | 0 | 0 | 0 | 0 | 0 | 0 | 0 | 0±0 |  | 0 | 0 | 0 | 0 | 0.0039 | 0 | 0 | 0.0005571±0.0014741 | 0.3559177 |
| Blautia wexlerae | 0 | 0 | 0 | 0.0119 | 0 | 0.0059 | 0 | 0.0025429  ±0.0046754 |  | 0 | 0.018 | 0 | 0 | 0.0116 | 0.0055 | 0.003 | 0.0054429 ±0.0069575 | 0.4536468 |
| Blautia_uc | 0.0028 | 0.0347 | 0 | 0 | 0.0219 | 0 | 0.02 | 0.0113429  ±0.0140885 |  | 0.0165 | 0.012 | 0 | 0.0079 | 0.0347 | 0 | 0.0475 | 0.0169429 ±0.0179331 | 0.3797887 |
| Brevibacillus borstelensis group | 0 | 0 | 0 | 0 | 0.0219 | 0 | 0 | 0.0031286  ±0.0082774 |  | 0 | 0 | 0 | 0 | 0 | 0 | 0 | 0±0 | 0.3559177 |
| Bradyrhizobium japonicum group | 0 | 0 | 0 | 0 | 0 | 0 | 0 | 0±0 |  | 0 | 0 | 0 | 0 | 0 | 0.0028 | 0 | 0.0004 ±0.0010583 | 0.3559177 |
| Brevibacillus borstelensis group | 0 | 0 | 0 | 0 | 0 | 0 | 0 | 0±0 |  | 0 | 0 | 0 | 0 | 0.0154 | 0 | 0 | 0.0022 ±0.0058207 | 0.3559177 |
| Brevundimonas vesicularis group | 0 | 0 | 0 | 0 | 0 | 0 | 0 | 0±0 |  | 0 | 0.003 | 0 | 0 | 0 | 0 | 0 | 0.0004286 ±0.0011339 | 0.3559177 |
| Butyricicoccus pullicaecorum | 0 | 0.1167 | 0 | 0 | 0 | 0 | 0 | 0.0166714  ±0.0441085 |  | 0 | 0.003 | 0 | 0 | 0 | 0 | 0 | 0.0004286 ±0.0011339 | 0.3559177 |
| Butyricicoccus_uc | 0 | 0.1577 | 0 | 0 | 0 | 0 | 0 | 0.0225286  ±0.059605 |  | 0 | 0.015 | 0 | 0 | 0 | 0 | 0 | 0.0021429 ±0.0056695 | 0.3559177 |
| CCFI_s | 0 | 0.0095 | 0 | 0 | 0 | 0 | 0.3172 | 0.0466714  ±0.1193444 |  | 0 | 0 | 0 | 0 | 0 | 0 | 0.003 | 0.0004286 ±0.0011339 | 0.3405749 |
| CP007496_s | 0 | 0 | 0 | 0.0024 | 0 | 0 | 0 | 0.0003429  ±0.0009071 |  | 0 | 0.003 | 0 | 0 | 0.0077 | 0 | 0 | 0.0015286 ±0.0029421 | 0.3746025 |
| CP017039_s | 0 | 0 | 0 | 0 | 0 | 0 | 0 | 0±0 |  | 0 | 0 | 0 | 0.0026 | 0 | 0 | 0 | 0.0003714 ±0.0009827 | 0.3559177 |
| CVTY_s | 0 | 0 | 0 | 0 | 0 | 0 | 0 | 0±0 |  | 0 | 0 | 0 | 0 | 0.0039 | 0 | 0 | 0.0005571 ±0.0014741 | 0.3559177 |
| Canibacter oris | 0 | 0 | 0.0049 | 0.0024 | 0 | 0 | 0 | 0.0010429  ±0.0019217 |  | 0 | 0 | 0 | 0.0026 | 0 | 0 | 0 | 0.0003714 ±0.0009827 | 0.3778825 |
| Citrobacter murliniae | 0 | 0 | 0 | 0 | 0.0031 | 0.0059 | 0 | 0.0012857  ±0.0023398 |  | 0 | 0 | 0 | 0 | 0 | 0.0028 | 0 | 0.0004 ±0.0010583 | 0.1723083 |
| Citrobacter portucalensis | 0 | 0 | 0 | 0 | 0 | 0.003 | 0 | 0.0004286  ±0.0011339 |  | 0 | 0 | 0.0026 | 0 | 0.0077 | 0 | 0 | 0.0014714 ±0.0029125 | 0.4419823 |
| Citrobacter_uc | 0 | 0 | 0 | 0 | 0 | 0 | 0.0033 | 0.0004714  ±0.0012473 |  | 0 | 0 | 0 | 0 | 0.0116 | 0 | 0 | 0.0016571 ±0.0043844 | 0.5338304 |
| Clostridioides difficile group | 0.0028 | 0.3154 | 0 | 0 | 0.1535 | 0.003 | 0 | 0.0678143  ±0.1230615 |  | 0 | 0.093 | 0 | 0 | 0.0386 | 0 | 0.3796 | 0.0730286 ±0.1395845 | 0.9431504 |
| Clostridioides_uc | 0 | 0 | 0 | 0.0142 | 0 | 0 | 0 | 0.0020286  ±0.0053671 |  | 0 | 0 | 0 | 0 | 0 | 0 | 0 | 0±0 | 0.3559177 |
| Clostridium celatum group | 0.0141 | 0.164 | 0 | 0 | 0.0063 | 0.1534 | 0.9782 | 0.188  ±0.3559338 |  | 0.0687 | 0 | 0 | 0 | 0 | 0.1053 | 0.2521 | 0.0608714 ±0.0943618 | 0.2636091 |
| Clostridium cocleatum | 0 | 0 | 0 | 0 | 0 | 0.003 | 0 | 0.0004286  ±0.0011339 |  | 0 | 0 | 0 | 0 | 0 | 0 | 0 | 0±0 | 0.3559177 |
| Clostridium clostridioforme group | 0 | 0 | 0 | 0 | 0 | 0 | 0 | 0±0 |  | 0 | 0.003 | 0 | 0 | 0 | 0 | 0 | 0.0004286 ±0.0011339 | 0.3559177 |
| Clostridium colinum | 0 | 0.1451 | 0.0025 | 0 | 0.0125 | 0.0413 | 0 | 0.0287714  ±0.0534285 |  | 0 | 0 | 0.0052 | 0.0158 | 0 | 0.2688 | 0.0653 | 0.0507286 ±0.0989785 | 0.6199128 |
| Clostridium glycyrrhizinilyticum | 0.0735 | 0.1199 | 0.0025 | 0 | 0 | 0 | 0 | 0.0279857  ±0.048823 |  | 0.1814 | 0.2009 | 0 | 0 | 0 | 0 | 0 | 0.0546143 ±0.0934414 | 0.1844218 |
| Clostridium hiranonis | 8.9336 | 0.0032 | 41.2256 | 38.9113 | 0 | 0 | 0 | 12.724814  ±18.973676 |  | 20.793 | 0.018 | 17.4132 | 0 | 0 | 0 | 0 | 5.4606 ±9.3705357 | 0.3159893 |
| Clostridium innocuum group | 0.0057 | 0 | 0.0025 | 0 | 0 | 0 | 0.0033 | 0.0016429  ±0.0022634 |  | 0.011 | 0 | 0 | 0.0423 | 0 | 0 | 0.0712 | 0.0177857 ±0.0281822 | 0.1742868 |
| Clostridium neonatale | 0 | 0 | 0.0074 | 0.2418 | 0 | 0 | 0.1836 | 0.0618286  ±0.1044602 |  | 0 | 0 | 0.2563 | 0.0132 | 0 | 0 | 0.0564 | 0.0465571±0.0947558 | 0.7919427 |
| Clostridium nexile | 0.3846 | 0.3596 | 0 | 0.2442 | 0.8335 | 0 | 1.8595 | 0.5259143  ±0.6523323 |  | 0.011 | 0.3269 | 0.0026 | 9.2441 | 1.5658 | 0 | 0.0059 | 1.5937571 ±3.4212987 | 0.4606882 |
| Clostridium paraputrificum | 0.017 | 0.0126 | 0.1186 | 0.0332 | 0.1159 | 0.1239 | 0.1636 | 0.0835429  ±0.0609769 |  | 0 | 0.009 | 0.0811 | 1.1489 | 0.1427 | 0.3963 | 1.5037 | 0.4688143 ±0.6091869 | 0.1339875 |
| Clostridium pasteurianum group | 0 | 0 | 0 | 0 | 0 | 0 | 0 | 0±0 |  | 0 | 0 | 0 | 0 | 0 | 0 | 0.003 | 0.0004286 ±0.0011339 | 0.3559177 |
| Clostridium perfringens | 0.3026 | 0.0063 | 0 | 0.0024 | 0 | 0.0738 | 0 | 0.0550143  ±0.112452 |  | 0 | 0 | 0 | 0 | 0 | 0.2328 | 0.2818 | 0.0735143 ±0.1263439 | 0.7945425 |
| Clostridium ramosum | 0.2432 | 0.2965 | 0.089 | 0.3485 | 0.7614 | 0 | 0.2804 | 0.2884286  ±0.2423663 |  | 0.0934 | 0.2879 | 0.034 | 0.6101 | 0.2121 | 0 | 0.4894 | 0.2467 ±0.2321855 | 0.6934352 |
| Clostridium spiroforme | 0.0255 | 0 | 0 | 0 | 1.5134 | 0 | 0 | 0.2198429  ±0.5704842 |  | 0 | 0 | 0 | 0 | 0.5553 | 0 | 0.0119 | 0.0810286 ±0.2091811 | 0.3487875 |
| Clostridium tertium group | 0.0255 | 0 | 0.0074 | 0 | 0.0219 | 0 | 0.1502 | 0.0292857  ±0.0543769 |  | 0 | 0.039 | 0.0078 | 0.0343 | 0.1465 | 0 | 0.0534 | 0.0401429 ±0.051303 | 0.6857021 |
| Clostridium_g24_uc | 0.0028 | 0 | 0 | 0 | 0 | 0 | 0 | 0.0004  ±0.0010583 |  | 0 | 0 | 0 | 0.0053 | 0 | 0 | 0 | 0.0007571±0.0020032 | 0.7092879 |
| Clostridium_uc | 0 | 0 | 0 | 0 | 0 | 0.003 | 0 | 0.0004286  ±0.0011339 |  | 0 | 0 | 0 | 0 | 0 | 0 | 0.0178 | 0.0025429 ±0.0067278 | 0.4550805 |
| Collinsella aerofaciens group | 0 | 0.0032 | 0.0025 | 0 | 0 | 0 | 0 | 0.0008143  ±0.0014053 |  | 0.0027 | 0 | 0 | 0 | 0 | 0 | 0 | 0.0003857 ±0.0010205 | 0.5794789 |
| Collinsella intestinalis group | 0.2291 | 0.5615 | 0.1458 | 0.2916 | 0 | 0 | 0 | 0.1754286  ±0.2076178 |  | 0.3297 | 3.8923 | 0.0889 | 1.7326 | 0 | 0 | 0 | 0.8633571 ±1.4748338 | 0.205699 |
| Coprococcus comes group | 0 | 0.0032 | 0 | 0 | 0 | 0.003 | 0 | 0.0008857  ±0.0015137 |  | 0 | 0 | 0.0026 | 0 | 0 | 0.0028 | 0 | 0.0007714 ±0.0013187 | 0.8631472 |
| Coprococcus_g2_uc | 0.0283 | 0 | 0 | 0 | 0 | 0 | 0 | 0.0040429  ±0.0106964 |  | 0.1044 | 0 | 0 | 0 | 0 | 0 | 0 | 0.0149143 ±0.0394595 | 0.3559177 |
| Corynebacterium canis | 0 | 0 | 0.0025 | 0 | 0 | 0 | 0 | 0.0003571  ±0.0009449 |  | 0 | 0 | 0 | 0 | 0 | 0 | 0 | 0±0 | 0.3559177 |
| Corynebacterium freiburgense | 0 | 0 | 0.0025 | 0.0024 | 0 | 0 | 0 | 0.0007  ±0.0011958 |  | 0 | 0 | 0 | 0 | 0 | 0 | 0 | 0±0 | 0.1724148 |
| Corynebacterium nuruki group | 0 | 0 | 0 | 0 | 0.0031 | 0 | 0 | 0.0004429 ±0.0011717 |  | 0 | 0 | 0 | 0 | 0 | 0 | 0 | 0±0 | 0.3559177 |
| Corynebacterium pseudotuberculosis group | 0 | 0 | 0 | 0 | 0.0031 | 0 | 0 | 0.0004429 ±0.0011717 |  | 0 | 0 | 0 | 0 | 0 | 0 | 0 | 0±0 | 0.3559177 |
| Corynebacterium xerosis group | 0 | 0.0032 | 0 | 0 | 0 | 0 | 0 | 0.0004571 ±0.0012095 |  | 0 | 0 | 0 | 0 | 0 | 0 | 0 | 0±0 | 0.3559177 |
| Cuneatibacter_uc | 0 | 0 | 0 | 0 | 0 | 0 | 0.0067 | 0.0009571 ±0.0025324 |  | 0 | 0 | 0 | 0 | 0 | 0 | 0 | 0±0 | 0.3559177 |
| Corynebacterium durum | 0 | 0 | 0 | 0 | 0 | 0 | 0 | 0±0 |  | 0 | 0 | 0 | 0 | 0 | 0 | 0.003 | 0.0004286 ±0.0011339 | 0.3559177 |
| Corynebacterium freiburgense | 0 | 0 | 0 | 0 | 0 | 0 | 0 | 0±0 |  | 0 | 0 | 0 | 0.0026 | 0 | 0 | 0 | 0.0003714 ±0.0009827 | 0.3559177 |
| Corynebacterium mustelae | 0 | 0 | 0 | 0 | 0 | 0 | 0 | 0±0 |  | 0 | 0 | 0 | 0.0026 | 0 | 0.0028 | 0 | 0.0007714 ±0.0013187 | 0.1726588 |
| Corynebacterium urealyticum | 0 | 0 | 0 | 0 | 0 | 0 | 0 | 0±0 |  | 0 | 0 | 0 | 0 | 0.0039 | 0 | 0 | 0.0005571 ±0.0014741 | 0.3559177 |
| Cutibacterium acnes group | 0 | 0 | 0 | 0 | 0 | 0 | 0 | 0±0 |  | 0 | 0.003 | 0 | 0 | 0 | 0 | 0 | 0.0004286 ±0.0011339 | 0.3559177 |
| DQ113748_s | 0 | 0.0126 | 0 | 0.0024 | 0 | 0 | 0 | 0.0021429 ±0.0046971 |  | 0 | 0.003 | 0 | 2.4484 | 0 | 0 | 0 | 0.3502 ±0.9252199 | 0.3579679 |
| DQ451512_s | 0 | 0 | 0 | 0 | 0.0031 | 0 | 0 | 0.0004429 ±0.0011717 |  | 0 | 0 | 0 | 0 | 0 | 0 | 0 | 0±0 | 0.3559177 |
| DQ456377_s | 0 | 0 | 0 | 0 | 0 | 0 | 0.0033 | 0.0004714 ±0.0012473 |  | 0 | 0.003 | 0 | 0 | 0 | 0.0028 | 0 | 0.0008286 ±0.0014162 | 0.6707047 |
| DQ796080_s | 0 | 0 | 0.0025 | 0 | 0 | 0 | 0 | 0.0003571 ±0.0009449 |  | 0 | 0 | 0 | 0 | 0 | 0 | 0 | 0±0 | 0.3559177 |
| DQ815838_s | 0 | 0 | 0 | 0 | 0 | 0 | 0 | 0±0 |  | 0 | 0 | 0 | 0 | 0.0039 | 0 | 0 | 0.0005571 ±0.0014741 | 0.3559177 |
| Dorea longicatena | 0 | 0 | 0 | 0 | 0 | 0 | 0 | 0±0 |  | 0 | 0 | 0 | 0 | 0.0039 | 0 | 0.003 | 0.0009857 ±0.0017034 | 0.1766322 |
| Dialister invisus | 0 | 0 | 0 | 0 | 0 | 0.003 | 0 | 0.0004286 ±0.0011339 |  | 0 | 0 | 0 | 0 | 0 | 0 | 0 | 0±0 | 0.3559177 |
| Dorea massiliensis | 0 | 0 | 0 | 0 | 0 | 0.003 | 0 | 0.0004286 ±0.0011339 |  | 0 | 0 | 0 | 0.0317 | 0.0039 | 0.0028 | 0 | 0.0054857 ±0.0116711 | 0.3015925 |
| EF097676_s | 0 | 0.0032 | 0 | 0 | 0 | 0 | 0 | 0.0004571 ±0.0012095 |  | 0 | 0 | 0 | 0 | 0 | 0 | 0 | 0±0 | 0.3559177 |
| EF399598_s | 0.7918 | 3.2774 | 0.0049 | 0 | 0.4073 | 0 | 0 | 0.6402 ±1.2017503 |  | 2.6902 | 7.5507 | 0 | 0.0026 | 1.4346 | 0 | 5.1756 | 2.4076714 ±2.9585186 | 0.0729682 |
| EF405084_s | 0.0057 | 0.0032 | 18.5174 | 20.0602 | 0 | 0 | 0 | 5.5123571 ±9.4216347 |  | 0.011 | 0.003 | 31.9186 | 1.4685 | 0 | 0 | 0 | 4.7715857 ±11.983165 | 0.8404355 |
| EU289064_s | 2.3557 | 0 | 1.0378 | 1.2044 | 0 | 0 | 0 | 0.6568429 ±0.9180444 |  | 0.2088 | 0 | 1.4932 | 0.1321 | 0 | 0 | 0 | 0.2620143 ±0.5492622 | 0.2905395 |
| EU462785_s | 0.0028 | 0 | 0 | 0 | 0 | 0 | 0 | 0.0004 ±0.0010583 |  | 0 | 0 | 0 | 0 | 0 | 0 | 0 | 0±0 | 0.3559177 |
| EU462939_s | 0 | 0 | 0 | 0 | 0 | 0 | 0.0033 | 0.0004714 ±0.0012473 |  | 0 | 0 | 0 | 0 | 0 | 0 | 0 | 0±0 | 0.3559177 |
| EU463003_s | 0 | 0 | 0 | 0 | 0.0031 | 0 | 0 | 0.0004429 ±0.0011717 |  | 0 | 0 | 0 | 0 | 0 | 0 | 0 | 0±0 | 0.3559177 |
| EU728720_s | 0 | 0 | 0 | 0.0024 | 0 | 0 | 0 | 0.0003429 ±0.0009071 |  | 0 | 0 | 0 | 0 | 0 | 0 | 0 | 0±0 | 0.3559177 |
| EU460192_s | 0 | 0 | 0 | 0 | 0 | 0 | 0 | 0±0 |  | 0 | 0 | 0 | 0.0264 | 0 | 0 | 0 | 0.0037714 ±0.0099783 | 0.3559177 |
| EU461309_s | 0 | 0 | 0 | 0 | 0 | 0 | 0 | 0±0 |  | 0 | 0 | 0.0026 | 0 | 0 | 0 | 0 | 0.0003714 ±0.0009827 | 0.3559177 |
| EU462857_s | 0 | 0 | 0 | 0 | 0 | 0 | 0 | 0±0 |  | 0 | 0 | 0 | 0 | 0.0039 | 0 | 0 | 0.0005571 ±0.0014741 | 0.3559177 |
| EU463003_s | 0 | 0 | 0 | 0 | 0 | 0 | 0 | 0±0 |  | 0 | 0 | 0 | 0 | 0 | 0 | 0.003 | 0.0004286 ±0.0011339 | 0.3559177 |
| EU506235_s | 0 | 0 | 0 | 0 | 0 | 0 | 0 | 0±0 |  | 0 | 0 | 0 | 0 | 0 | 0 | 0.003 | 0.0004286 ±0.0011339 | 0.3559177 |
| EU509323_s | 0 | 0 | 0 | 0 | 0 | 0 | 0 | 0±0 |  | 0 | 0 | 0 | 0 | 0 | 0.0028 | 0 | 0.0004 ±0.0010583 | 0.3559177 |
| EU728773_s | 0 | 0 | 0 | 0 | 0 | 0.003 | 0 | 0.0004286 ±0.0011339 |  | 0 | 0 | 0 | 0 | 0 | 0 | 0.0089 | 0.0012714 ±0.0033639 | 0.5712589 |
| EU772680_s | 0 | 0 | 0.042 | 0.0213 | 0 | 0 | 0 | 0.0090429 ±0.0165594 |  | 0 | 0 | 0.0523 | 0.4596 | 0 | 0 | 0 | 0.0731286 ±0.1715289 | 0.3439232 |
| EU772717_s | 0.0396 | 0 | 0.0025 | 0.0024 | 0 | 0 | 0 | 0.0063571 ±0.0147042 |  | 0 | 0 | 0.0471 | 0 | 0 | 0 | 0 | 0.0067286 ±0.0178021 | 0.9691373 |
| EU772949_s | 0.0028 | 0 | 0.0049 | 0 | 0 | 0 | 0 | 0.0011 ±0.001974 |  | 0 | 0 | 0.0052 | 0 | 0 | 0.0028 | 0 | 0.0011429 ±0.0020711 | 0.9464916 |
| EU774287_s | 0 | 0 | 0 | 0 | 0 | 0.0059 | 0 | 0.0008429 ±0.00223 |  | 0 | 0 | 0 | 0 | 0 | 0 | 0.003 | 0.0004286 ±0.0011339 | 0.6951286 |
| EU776034_s | 0 | 0 | 0 | 0 | 0 | 0 | 0 | 0±0 |  | 0 | 0 | 0 | 0 | 0.0039 | 0 | 0 | 0.0005571 ±0.0014741 | 0.3559177 |
| EU776475_s | 0 | 0 | 0 | 0 | 0 | 0 | 0 | 0±0 |  | 0 | 0 | 0 | 0 | 0 | 0 | 0.003 | 0.0004286 ±0.0011339 | 0.3559177 |
| EU778993_s | 0 | 0 | 0 | 0 | 0 | 0.2862 | 0 | 0.0408857 ±0.1081734 |  | 0.0027 | 0 | 0.0026 | 0 | 0 | 0 | 0 | 0.0007571 ±0.0012934 | 0.3656633 |
| EU779209_s | 0 | 0 | 0 | 0 | 0 | 0 | 0 | 0±0 |  | 0 | 0 | 0 | 0.0026 | 0 | 0 | 0 | 0.0003714 ±0.0009827 | 0.3559177 |
| EU845632_s | 0.0028 | 0 | 0 | 0 | 0 | 0 | 0 | 0.0004 ±0.0010583 |  | 0 | 0 | 0 | 0 | 0 | 0 | 0 | 0±0 | 0.3559177 |
| Eisenbergiella_uc | 0.0028 | 0 | 0 | 0 | 0 | 0 | 0 | 0.0004 ±0.0010583 |  | 0 | 0 | 0 | 0 | 0 | 0 | 0 | 0±0 | 0.3559177 |
| Enterobacter_uc | 0 | 0 | 0.0025 | 0 | 0.0595 | 0 | 0 | 0.0088571 ±0.0223508 |  | 0 | 0 | 0 | 0 | 0.0154 | 0 | 0 | 0.0022 ±0.0058207 | 0.3278146 |
| Enterobacteriaceae group | 0.0424 | 0.0095 | 0 | 0 | 0.1473 | 0 | 0 | 0.0284571 ±0.0546453 |  | 0.0027 | 0.003 | 0 | 0 | 0.1311 | 0 | 0 | 0.0195429 ±0.0492105 | 0.1635246 |
| Enterococcus faecalis | 0 | 0 | 0 | 0 | 0.0094 | 0.003 | 0 | 0.0017714 ±0.0035448 |  | 0 | 0 | 0 | 0 | 0.4319 | 0 | 0 | 0.0617 ±0.1632429 | 0.3596365 |
| Enterococcus faecium group | 0 | 0 | 0 | 0 | 0 | 0.0236 | 0.03 | 0.0076571 ±0.0132069 |  | 0 | 0 | 0 | 0 | 0 | 0.0055 | 0.3826 | 0.0554429 ±0.1442773 | 0.3837686 |
| Enterorhabdus caecimuris | 0 | 0 | 0 | 0 | 0 | 0 | 0 | 0±0 |  | 0 | 0 | 0 | 0 | 0.0039 | 0 | 0 | 0.0005571 ±0.1442773 | 0.3559177 |
| Escherichia albertii | 0 | 0 | 0 | 0 | 0 | 0 | 0 | 0±0 |  | 0 | 0 | 0 | 0 | 0 | 0 | 0.003 | 0.0004286 ±0.0011339 | 0.3559177 |
| Enterococcus italicus group | 0 | 0 | 0 | 0 | 0.0031 | 0 | 0 | 0.0004429 ±0.0011717 |  | 0 | 0 | 0 | 0 | 0 | 0 | 0 | 0±0 | 0.3559177 |
| Epulopiscium_uc | 0 | 0 | 0 | 0 | 0 | 0 | 0.0033 | 0.0004714 ±0.0012473 |  | 0 | 0 | 0 | 0 | 0 | 0 | 0 | 0±0 | 0.3559177 |
| Escherichia coli group | 3.4982 | 0.6782 | 0.0914 | 0.0119 | 11.6152 | 4.5911 | 16.5454 | 5.2902 ±6.410196 |  | 0.9398 | 1.0285 | 0.1307 | 0 | 8.4535 | 6.28 | 3.8231 | 2.9508 ±3.329758 | 0.2519323 |
| Escherichia_uc | 0.0028 | 0 | 0 | 0 | 0.0063 | 0 | 0 | 0.0013 ±0.0024393 |  | 0 | 0 | 0 | 0 | 0.0077 | 0 | 0 | 0.0011 ±0.0029103 | 0.6890522 |
| Eubacterium dolichum | 0.0141 | 0.0284 | 0 | 0 | 0.094 | 0 | 0 | 0.0195 ±0.034592 |  | 0.0082 | 0.081 | 0 | 0 | 0.4011 | 0 | 0.0208 | 0.0730143 ±0.1475491 | 0.2592771 |
| Eubacterium nodatum | 0 | 0 | 0 | 0.0024 | 0 | 0 | 0 | 0.0003429 ±0.0009071 |  | 0 | 0 | 0 | 0 | 0 | 0 | 0 | 0±0 | 0.3559177 |
| Eubacterium ramulus | 0 | 0 | 0.0025 | 0 | 0 | 0 | 0 | 0.0003571 ±0.0009449 |  | 0 | 0 | 0 | 0 | 0 | 0 | 0 | 0±0 | 0.3559177 |
| Eubacterium saphenum | 0 | 0 | 0 | 0.0024 | 0 | 0 | 0 | 0.0003429 ±0.0009071 |  | 0 | 0 | 0 | 0 | 0 | 0 | 0 | 0±0 | 0.3559177 |
| Eubacterium eligens group | 0 | 0 | 0 | 0 | 0 | 0 | 0 | 0±0 |  | 0 | 0 | 0.0026 | 0 | 0 | 0 | 0 | 0.0003714 ±0.0009827 | 0.3559177 |
| Eubacterium hallii | 0 | 0 | 0 | 0 | 0 | 0 | 0 | 0±0 |  | 0 | 0.006 | 0 | 0 | 0 | 0 | 0.003 | 0.0012857 ±0.0023604 | 0.1996217 |
| Exiguobacterium acetylicum group | 0 | 0 | 0 | 0 | 0 | 0 | 0 | 0±0 |  | 0 | 0 | 0 | 0.0026 | 0 | 0 | 0 | 0.0003714 ±0.0009827 | 0.3559177 |
| FCEY_s | 0.3309 | 0.0379 | 0.0766 | 0 | 0.0031 | 0.003 | 0.2804 | 0.1045571 ±0.1407813 |  | 0.2253 | 0 | 0.2746 | 2.7019 | 0.0154 | 0 | 0.086 | 0.4718857 ±0.9895562 | 0.3846049 |
| FJ152825_s | 0 | 0 | 0 | 0 | 0 | 0 | 0 | 0±0 |  | 0 | 0 | 0 | 0 | 0.0039 | 0 | 0 | 0.0005571 ±0.0014741 | 0.3559177 |
| FJ680388_s | 0 | 0 | 0 | 0 | 0 | 0 | 0 | 0±0 |  | 0 | 0 | 0 | 0 | 0 | 0.0028 | 0 | 0.0004 ±0.0010583 | 0.3559177 |
| FJ711049_s | 0 | 0 | 0 | 0 | 0 | 0 | 0 | 0±0 |  | 0 | 0 | 0 | 0 | 0 | 0 | 0.003 | 0.0004286 ±0.0011339 | 0.3559177 |
| FJ825526_s | 0 | 0 | 0.0025 | 0 | 0.0031 | 0 | 0.0033 | 0.0012714 ±0.0016039 |  | 0.0027 | 0 | 0 | 0.0053 | 0 | 0 | 0.0059 | 0.0019857±0.0026642 | 0.5534736 |
| FJTZ_s | 0 | 0 | 0 | 0 | 0.0094 | 0 | 0 | 0.0013429 ±0.0035529 |  | 0 | 0 | 0 | 0 | 0 | 0 | 0 | 0±0 | 0.3559177 |
| FM875685_s | 0 | 0 | 0 | 0 | 0 | 0 | 0 | 0±0 |  | 0 | 0.006 | 0 | 0 | 0 | 0 | 0 | 0.0008571 ±0.0022678 | 0.3559177 |
| FQLR_s | 0.0255 | 0 | 0 | 0 | 0 | 0 | 0 | 0.0036429 ±0.0096381 |  | 0.0632 | 0 | 0 | 0 | 0 | 0 | 0 | 0.0090286 ±0.0238874 | 0.3559177 |
| FWNZ_s | 0.017 | 0 | 0 | 0 | 0 | 0.003 | 0 | 0.0028571 ±0.0063358 |  | 0 | 0 | 0 | 0 | 0 | 0 | 0 | 0±0 | 0.2778646 |
| Faecalibacterium prausnitzii group | 1.6798 | 0 | 0 | 0.0024 | 0.0063 | 0.0059 | 0 | 0.2420571 ±0.6339909 |  | 2.0801 | 0.009 | 0 | 0 | 0.0077 | 0.0055 | 0.003 | 0.3007571 ±0.7846243 | 0.3424131 |
| Faecalibaculum rodentium | 0 | 0 | 0 | 0 | 0.0063 | 0.0059 | 0.0067 | 0.0027 ±0.0033754 |  | 0 | 0 | 0 | 0 | 0.0231 | 0.0028 | 0.003 | 0.0041286 ±0.0084768 | 0.607028 |
| Faecalicatena contorta group | 0.0028 | 0 | 0 | 0 | 0 | 0 | 0.0033 | 0.0008714 ±0.0014952 |  | 0 | 0 | 0 | 0.0317 | 0 | 0 | 0 | 0.0045286 ±0.0119815 | 0.4665753 |
| Faecalimonas umbilicata | 0.4723 | 2.2333 | 1.1515 | 0.0119 | 5.2671 | 5.2697 | 0.9348 | 2.1915143 ±2.2099614 |  | 0.0879 | 36.362 | 0.9048 | 14.6849 | 8.2993 | 86.6031 | 1.9338 | 21.267971 ±31.47776 | 0.1452766 |
| Faecalimonas_uc | 0 | 0 | 0 | 0 | 0 | 0 | 0 | 0±0 |  | 0 | 0.012 | 0 | 0 | 0 | 0 | 0 | 0.0017143 ±0.0045356 | 0.3559177 |
| Flavonifractor plautii | 1.0152 | 0 | 0 | 0 | 0.5671 | 0 | 0.1335 | 0.2451143 ±0.3978041 |  | 0.1346 | 0.003 | 0 | 0.0026 | 0.6633 | 0 | 0 | 0.1147857 ±0.2469377 | 0.3464163 |
| Flintibacter butyricus group | 0 | 0 | 0 | 0 | 0.0063 | 0 | 0 | 0.0009 ±0.0023812 |  | 0 | 0 | 0 | 0 | 0.0039 | 0 | 0 | 0.0005571 ±0.0014741 | 0.3559177 |
| Fournierella massiliensis group | 0.0311 | 0 | 0 | 0 | 0 | 0 | 0 | 0.0044429 ±0.0117547 |  | 0.0137 | 0 | 0 | 0 | 0 | 0 | 0 | 0.0019571 ±0.0051781 | 0.3559177 |
| Fusicatenibacter saccharivorans | 0 | 0 | 0.0025 | 0.0047 | 0.0063 | 0 | 0 | 0.0019286 ±0.0026456 |  | 0.0027 | 0.009 | 0.0026 | 0 | 0 | 0 | 0 | 0.0020429 ±0.0033125 | 0.9537401 |
| Fusobacterium necrogenes group | 10.1411 | 7.4569 | 0.1903 | 0.1849 | 0 | 0 | 0 | 2.5676 ±4.3276183 |  | 0.3352 | 0.039 | 0.4184 | 0.4437 | 0 | 0 | 0 | 0.1766143 ±0.2111319 | 0.1922495 |
| Fusobacterium perfoetens | 0.0028 | 0 | 0.2842 | 3.8622 | 0 | 0 | 0 | 0.5927429 ±1.4455655 |  | 0 | 0.003 | 2.058 | 0.0026 | 0 | 0 | 0 | 0.2948 ±0.7774992 | 0.6599459 |
| Fusobacterium periodonticum group | 0 | 0 | 0 | 0 | 0 | 0 | 0 | 0±0 |  | 0 | 0.003 | 0 | 0 | 0 | 0 | 0 | 0.0004286 ±0.0011339 | 0.3559177 |
| Fusobacterium varium group | 0.0141 | 0.0032 | 8.2308 | 6.2663 | 0 | 0 | 0 | 2.0734857 ±3.5804421 |  | 0.0055 | 0.006 | 14.4217 | 16.576 | 0 | 0 | 0 | 4.4298857 ±7.587073 | 0.1880754 |
| Fusobacterium_uc | 0 | 0 | 0.0766 | 0.0593 | 0 | 0 | 0 | 0.0194143 ±0.0335302 |  | 0 | 0 | 0.2824 | 0.2377 | 0 | 0 | 0 | 0.0743 ±0.1275458 | 0.1736064 |
| GQ354986_s | 0 | 0 | 0 | 0 | 0 | 0.003 | 0 | 0.0004286 ±0.0011339 |  | 0 | 0 | 0 | 0 | 0 | 0 | 0 | 0±0 | 0.3559177 |
| GQ082178_s | 0 | 0 | 0 | 0 | 0 | 0 | 0 | 0±0 |  | 0.0055 | 0 | 0 | 0 | 0 | 0 | 0 | 0.0007857 ±0.0020788 | 0.3559177 |
| Gemella palaticanis | 0 | 0 | 0 | 0 | 0 | 0 | 0 | 0±0 |  | 0 | 0 | 0 | 0 | 0 | 0.0028 | 0 | 0.0004 ±0.0010583 | 0.3559177 |
| Gemmiger formicilis group | 0 | 0 | 0 | 0.0024 | 0 | 0 | 0.0033 | 0.0008143 ±0.0014147 |  | 0 | 0 | 0 | 0 | 0 | 0.0083 | 0 | 0.0011857 ±0.0031371 | 0.8022707 |
| HM831097_s | 0.0707 | 0.0284 | 0 | 0 | 0 | 0 | 0.0634 | 0.0232143 ±0.0317603 |  | 0 | 0 | 0 | 0 | 0 | 0 | 0 | 0±0 | 0.1013037 |
| HM124343_s | 0 | 0 | 0 | 0 | 0 | 0 | 0 | 0±0 |  | 0 | 0 | 0 | 0 | 0 | 0 | 0.003 | 0.0004286 0.0011339 | 0.3559177 |
| HM558072_s | 0 | 0 | 0 | 0 | 0 | 0 | 0 | 0±0 |  | 0 | 0 | 0 | 0 | 0.0039 | 0 | 0 | 0.0005571 ±0.0014741 | 0.3559177 |
| HM831097_s | 0 | 0 | 0 | 0 | 0 | 0 | 0 | 0±0 |  | 0.011 | 0 | 0 | 0 | 0.0347 | 0 | 0 | 0.0065286 ±0.0130814 | 0.2348244 |
| HQ772845_s | 0 | 0 | 0.0074 | 0.0119 | 0 | 0 | 0 | 0.0027571 ±0.0048846 |  | 0 | 0 | 0.0758 | 0 | 0 | 0 | 0 | 0.0108286 ±0.0286497 | 0.4586175 |
| HQ781386_s | 0.0028 | 0 | 0 | 0 | 0 | 0 | 0 | 0.0004 ±0.0010583 |  | 0 | 0 | 0 | 0 | 0 | 0 | 0 | 0±0 | 0.3559177 |
| HQ782813_s | 0.0424 | 0 | 0.0025 | 0 | 0 | 0 | 0 | 0.0064143 ±0.0158955 |  | 0 | 0 | 0.0235 | 0 | 0 | 0 | 0 | 0.0033571 ±0.0088822 | 0.6856644 |
| Haemophilus parainfluenzae group | 0 | 0 | 0 | 0 | 0 | 0 | 0 | 0±0 |  | 0 | 0.006 | 0 | 0 | 0 | 0 | 0 | 0.0008571 ±0.0022678 | 0.3559177 |
| Haemophilus sputorum | 0 | 0 | 0 | 0 | 0 | 0 | 0 | 0±0 |  | 0 | 0.003 | 0 | 0 | 0 | 0 | 0 | 0.0004286 ±0.0011339 | 0.3559177 |
| Helicobacter canicola | 0 | 0.1325 | 0 | 0 | 0 | 0.7376 | 0 | 0.1243 ±0.2749111 |  | 0 | 0 | 0 | 0 | 0.0039 | 0.0028 | 0 | 0.0009571 0.0016652 | 0.2788936 |
| Helicobacter canis group | 0 | 0 | 0 | 0 | 0 | 0.0708 | 0 | 0.0101143 ±0.0267599 |  | 0 | 0 | 0 | 0 | 0 | 0 | 0 | 0±0 | 0.3559177 |
| Helicobacter cinaedi | 0 | 0 | 0 | 0 | 0 | 0.003 | 0 | 0.0004286 ±0.0011339 |  | 0 | 0 | 0 | 0 | 0 | 0 | 0 | 0±0 | 0.3559177 |
| Helicobacter_uc | 0 | 0.0032 | 0 | 0 | 0 | 0.003 | 0 | 0.0008857 ±0.0015137 |  | 0 | 0 | 0 | 0 | 0 | 0 | 0 | 0±0 | 0.1725742 |
| Intestinibacter bartlettii | 0 | 0 | 0 | 0 | 0.0031 | 0 | 0 | 0.0004429 ±0.0011717 |  | 0 | 0 | 0 | 0 | 0 | 0 | 0 | 0±0 | 0.3559177 |
| Hungatella_uc | 0 | 0 | 0 | 0 | 0 | 0 | 0 | 0±0 |  | 0 | 0 | 0 | 0 | 0 | 0 | 0.003 | 0.0004286 ±0.0011339 | 0.3559177 |
| JF241012_s | 0 | 0.0063 | 0 | 0 | 0 | 0 | 0 | 0.0009 ±0.0023812 |  | 0 | 0.003 | 0 | 0.0026 | 0.0039 | 0 | 0.003 | 0.0017857 ±0.0017151 | 0.3779395 |
| JH815513_s | 4.3409 | 0 | 0 | 0.946 | 0 | 0 | 0 | 0.7552714 ±1.6199426 |  | 2.2203 | 0 | 0 | 0.0581 | 0 | 0 | 0 | 0.3254857 ±0.8358151 | 0.2127534 |
| JN178768_s | 0 | 0 | 0 | 0 | 0 | 0 | 0 | 0±0 |  | 0 | 0.003 | 0 | 0 | 0 | 0 | 0 | 0.0004286 ±0.0011339 | 0.3559177 |
| JN713479_s | 0 | 0 | 0 | 0 | 0 | 0 | 0 | 0±0 |  | 0 | 0 | 0 | 0.0026 | 0 | 0 | 0 | 0.0003714 ±0.0009827 | 0.3559177 |
| JN713494_s | 0 | 0 | 0 | 0 | 0 | 0 | 0 | 0±0 |  | 0 | 0 | 0 | 0.0026 | 0 | 0 | 0 | 0.0003714 ±0.0009827 | 0.3559177 |
| JN713548_s | 0 | 0 | 0 | 0 | 0 | 0 | 0 | 0±0 |  | 0 | 0.006 | 0 | 0.0106 | 0 | 0 | 0 | 0.0023714 ±0.0042621 | 0.1914162 |
| JN713416_s | 0 | 0 | 0.0049 | 0 | 0 | 0 | 0 | 0.0007 ±0.001852 |  | 0 | 0 | 0 | 0 | 0 | 0 | 0 | 0±0 | 0.3559177 |
| JN713466_s | 0 | 0 | 0 | 0.0024 | 0 | 0 | 0 | 0.0003429 ±0.0009071 |  | 0 | 0 | 0 | 0 | 0 | 0 | 0 | 0±0 | 0.3559177 |
| JX198565_s | 0 | 0 | 0 | 0 | 0 | 0 | 0 | 0±0 |  | 0 | 0 | 0 | 0 | 0.0039 | 0 | 0 | 0.0005571 ±0.0014741 | 0.3559177 |
| KB822463_s | 0 | 0 | 0 | 0 | 0 | 0 | 0 | 0±0 |  | 0 | 0 | 0 | 0 | 0 | 0.0028 | 0 | 0.0004 ±0.0010583 | 0.3559177 |
| KE159567_s | 0 | 0 | 0 | 0 | 0.0031 | 0 | 0 | 0.0004429 ±0.0011717 |  | 0 | 0 | 0 | 0 | 0.0077 | 0.0055 | 0.003 | 0.0023143 ±0.0031898 | 0.0893606 |
| KE159600_s | 0 | 0 | 0 | 0 | 0.0031 | 0 | 0 | 0.0004429 ±0.0011717 |  | 0 | 0 | 0 | 0 | 0 | 0.0028 | 0 | 0.0004 ±0.0010583 | 0.9491299 |
| KE159605_s | 0 | 0 | 0 | 0 | 0.0031 | 0.003 | 0 | 0.0008714 ±0.0014885 |  | 0 | 0 | 0.0026 | 0 | 0 | 0 | 0 | 0.0003714 ±0.0009827 | 0.5297295 |
| KE159810_s | 0 | 0 | 0 | 0 | 0 | 0.003 | 0 | 0.0004286 ±0.0011339 |  | 0 | 0 | 0 | 0 | 0 | 0 | 0 | 0±0 | 0.3559177 |
| KE159605_s | 0 | 0 | 0 | 0 | 0 | 0 | 0 | 0±0 |  | 0 | 0 | 0 | 0 | 0 | 0 | 0.003 | 0.0004286 ±0.0011339 | 0.3559177 |
| KE159797_g_uc | 0 | 0 | 0 | 0 | 0 | 0 | 0 | 0±0 |  | 0 | 0 | 0 | 0 | 0 | 0.0055 | 0 | 0.0007857 ±0.0020788 | 0.3559177 |
| KI535319_s | 0 | 0 | 0 | 0 | 0 | 0 | 0 | 0±0 |  | 0 | 0 | 0 | 0 | 0 | 0.0028 | 0.0059 | 0.0012429 ±0.0023035 | 0.2033435 |
| KV831974_s group | 0 | 0 | 0 | 0 | 0 | 0 | 0 | 0±0 |  | 0 | 0.003 | 0 | 0 | 0 | 0 | 0 | 0.0004286 ±0.0011339 | 0.3559177 |
| Kineothrix alysoides | 0 | 0 | 0 | 0 | 0.0031 | 0 | 0 | 0.0004429 ±0.0011717 |  | 0 | 0 | 0 | 0 | 0 | 0.0028 | 0 | 0.0004 ±0.0010583 | 0.9491299 |
| Kurthia zopfii group | 0 | 0 | 0 | 0 | 0 | 0 | 0 | 0±0 |  | 0 | 0 | 0 | 0.0026 | 0 | 0 | 0 | 0.0003714 ±0.0009827 | 0.3559177 |
| Kocuria kristinae | 0 | 0 | 0 | 0 | 0.0031 | 0 | 0 | 0.0004429 ±0.0011717 |  | 0 | 0 | 0 | 0 | 0 | 0 | 0 | 0±0 | 0.3559177 |
| Kroppenstedtia eburnea | 0 | 0 | 0 | 0 | 0.0219 | 0 | 0 | 0.0031286 ±0.0082774 |  | 0 | 0 | 0 | 0 | 0 | 0 | 0 | 0±0 | 0.3559177 |
| LN879468_s | 0 | 0.8075 | 0 | 0 | 0.0251 | 0 | 0.671 | 0.2148 ±0.3605445 |  | 0.0055 | 0 | 0 | 0 | 0 | 0 | 7.314 | 1.0456429 ±2.7640864 | 0.426962 |
| LN913006_s group | 0 | 0 | 0 | 0 | 0 | 0 | 0.0033 | 0.0004714 ±0.0012473 |  | 0 | 0.006 | 0 | 0 | 0.0039 | 0.0055 | 0 | 0.0022 ±0.002816 | 0.2332586 |
| LT635469_s | 0 | 0.0032 | 0 | 0 | 0.0251 | 0 | 0.0367 | 0.0092857 ±0.0151853 |  | 0 | 0.006 | 0 | 0.037 | 0.0077 | 0 | 0.0326 | 0.0119 ±0.0159989 | 0.6909951 |
| LT907848_s | 0 | 0 | 0 | 0 | 0 | 0 | 0 | 0±0 |  | 0.0027 | 0 | 0 | 0 | 0 | 0 | 0 | 0.0003857 ±0.0010205 | 0.3559177 |
| Lactobacillus brevis | 0 | 0 | 0 | 0 | 0 | 0 | 0 | 0±0 |  | 0 | 0.018 | 0 | 0 | 0 | 0 | 0 | 0.0025714 ±0.0068034 | 0.3559177 |
| Lactobacillus fermentum | 0 | 0 | 0 | 0 | 0.0031 | 0 | 0 | 0.0004429 ±0.0011717 |  | 0 | 0 | 0 | 0 | 0 | 0 | 0 | 0±0 | 0.3559177 |
| Lactobacillus gasseri group | 0 | 0 | 0 | 0 | 0 | 0.0089 | 0.0167 | 0.0036571 ±0.0066392 |  | 0 | 0 | 0 | 0 | 0.0116 | 0.0139 | 0.0059 | 0.0044857 ±0.006079 | 0.7555287 |
| Lactobacillus helveticus group | 0 | 0 | 0 | 0 | 0 | 0.003 | 0 | 0.0004286 ±0.0011339 |  | 0 | 0 | 0 | 0 | 0 | 0 | 0 | 0±0 | 0.3559177 |
| Lactobacillus intestinalis | 0 | 0 | 0 | 0 | 0.0031 | 0 | 0.0067 | 0.0014 ±0.002607 |  | 0 | 0 | 0 | 0 | 0.0116 | 0 | 0.0059 | 0.0025 ±0.0045757 | 0.4086293 |
| Lactobacillus murinus group | 0 | 0 | 0 | 0 | 0.0125 | 0.0148 | 0.0534 | 0.0115286 ±0.019564 |  | 0 | 0 | 0 | 0 | 0.0309 | 0.0111 | 0.0119 | 0.0077 ±0.0115802 | 0.5965513 |
| Lactobacillus paracasei group | 0 | 0 | 0 | 0 | 0.0031 | 0 | 0 | 0.0004429 ±0.0011717 |  | 0 | 0 | 0 | 0 | 0 | 0 | 0 | 0±0 | 0.3559177 |
| Lactobacillus plantarum group | 0 | 0 | 0 | 0 | 0 | 0.003 | 0 | 0.0004286 ±0.0011339 |  | 0 | 0.015 | 0 | 0 | 0 | 0 | 0 | 0.0021429 ±0.0056695 | 0.4757861 |
| Lactobacillus reuteri group | 0 | 0 | 0 | 0 | 0.0031 | 0.0059 | 0.01 | 0.0027143 ±0.0039338 |  | 0 | 0 | 0 | 0 | 0 | 0 | 0.003 | 0.0004286 ±0.0011339 | 0.0970664 |
| Lactobacillus rogosae group | 0 | 0 | 0 | 0 | 0 | 0 | 0.0033 | 0.0004714 ±0.0012473 |  | 0 | 0.003 | 0 | 0 | 0 | 0 | 0 | 0.0004286 ±0.0011339 | 0.9523493 |
| Lactobacillus sakei group | 0 | 0 | 0 | 0 | 0 | 0 | 0 | 0±0 |  | 0 | 0.09 | 0 | 0 | 0 | 0 | 0 | 0.0128571 ±0.0340168 | 0.3559177 |
| Lactococcus lactis group | 0 | 0.0032 | 0 | 0 | 0.0031 | 0.003 | 0 | 0.0013286 ±0.001658 |  | 0 | 0.015 | 0 | 0 | 0 | 0 | 0.0059 | 0.0029857 ±0.005736 | 0.4459537 |
| Lactonifactor longoviformis | 0 | 0 | 0 | 0 | 0 | 0 | 0 | 0±0 |  | 0.022 | 0 | 0 | 0 | 0 | 0 | 0 | 0.0031429 ±0.0083152 | 0.3559177 |
| Lautropia mirabilis | 0 | 0 | 0 | 0 | 0 | 0 | 0 | 0±0 |  | 0 | 0.003 | 0 | 0 | 0 | 0 | 0 | 0.0004286 ±0.0011339 | 0.3559177 |
| Lysinibacillus xylanilyticus group | 0 | 0 | 0.0049 | 0 | 0.0063 | 0 | 0 | 0.0016 ±0.0027622 |  | 0 | 0 | 0 | 0 | 0.0039 | 0 | 0 | 0.0005571 ±0.0014741 | 0.2010756 |
| Macrococcus equipercicus group | 0 | 0 | 0 | 0 | 0.0501 | 0 | 0 | 0.0071571 ±0.018936 |  | 0 | 0 | 0 | 0 | 0.027 | 0 | 0 | 0.0038571 ±0.010205 | 0.3559177 |
| Massilia arvi group | 0 | 0 | 0 | 0 | 0 | 0 | 0 | 0±0 |  | 0 | 0.003 | 0 | 0 | 0 | 0 | 0 | 0.0004286 ±0.0011339 | 0.3559177 |
| Megamonas rupellensis group | 1.2613 | 0.0095 | 17.2795 | 2.6744 | 0.0094 | 66.5083 | 0.0134 | 12.536543 ±24.598524 |  | 0.7337 | 0.009 | 19.4712 | 1.0934 | 0.0116 | 0.0222 | 0.0089 | 3.05 ±7.2542488 | 0.3569927 |
| Megamonas_uc | 0 | 0 | 0.0297 | 0 | 0 | 2.1952 | 0 | 0.3178429 ±0.8279107 |  | 0.0027 | 0 | 0.0758 | 0.0026 | 0 | 0 | 0 | 0.0115857±0.0283434 | 0.3683182 |
| Mucispirillum schaedleri | 0 | 0 | 0 | 0 | 0.0031 | 0.003 | 0 | 0.0008714 ±0.0014885 |  | 0 | 0 | 0 | 0 | 0 | 0 | 0 | 0±0 | 0.172377 |
| Murimonas intestini | 0 | 0 | 0 | 0 | 0.0031 | 0 | 0 | 0.0004429 ±0.0011717 |  | 0 | 0 | 0 | 0 | 0 | 0 | 0 | 0±0 | 0.3559177 |
| Oceanobacillus caeni | 0 | 0 | 0 | 0 | 0.0063 | 0 | 0 | 0.0009 ±0.0023812 |  | 0 | 0 | 0 | 0 | 0 | 0 | 0 | 0±0 | 0.3559177 |
| Morganella morganii group | 0 | 0 | 0 | 0 | 0 | 0 | 0 | 0±0 |  | 0.0027 | 0 | 0 | 0 | 0 | 0 | 0 | 0.0003857 ±0.0010205 | 0.3559177 |
| Muricomes intestini group | 0 | 0 | 0 | 0 | 0 | 0 | 0 | 0±0 |  | 0 | 0 | 0 | 0 | 0 | 0.0028 | 0 | 0.0004 ±0.0010583 | 0.3559177 |
| NFKJ_s | 0 | 0 | 0 | 0 | 0 | 0 | 0 | 0±0 |  | 0 | 0.003 | 0 | 0 | 0 | 0.0055 | 0 | 0.0012143 ±0.0021958 | 0.1937598 |
| NFLD_s | 0 | 0 | 0 | 0 | 0 | 0 | 0 | 0±0 |  | 0 | 0 | 0 | 0 | 0 | 0.0028 | 0 | 0.0004 ±0.0010583 | 0.3559177 |
| Neisseria perflava | 0 | 0 | 0 | 0 | 0 | 0 | 0 | 0±0 |  | 0 | 0 | 0 | 0 | 0 | 0.0028 | 0 | 0.0004 ±0.0010583 | 0.3559177 |
| Nesterenkonia_uc | 0 | 0 | 0 | 0 | 0 | 0 | 0 | 0±0 |  | 0 | 0 | 0 | 0.0185 | 0 | 0 | 0 | 0.0026429 ±0.0069923 | 0.3559177 |
| PAC000025_s | 0 | 0 | 0 | 0.0024 | 0 | 0 | 0 | 0.0003429 ±0.0009071 |  | 0 | 0 | 0 | 0 | 0 | 0 | 0 | 0±0 | 0.3559177 |
| PAC000739_s | 0 | 0 | 0 | 0 | 0 | 0.003 | 0 | 0.0004286 ±0.0011339 |  | 0 | 0 | 0 | 0 | 0 | 0 | 0 | 0±0 | 0.3559177 |
| PAC001068_s | 0 | 0 | 0 | 0 | 0 | 0.003 | 0 | 0.0004286 ±0.0011339 |  | 0 | 0 | 0 | 0 | 0 | 0 | 0 | 0±0 | 0.3559177 |
| PAC000194_s | 0 | 0 | 0 | 0 | 0 | 0 | 0 | 0±0 |  | 0 | 0 | 0 | 0.0026 | 0 | 0 | 0 | 0.0003714 ±0.0009827 | 0.3559177 |
| PAC000479_s | 0 | 0 | 0 | 0 | 0 | 0 | 0 | 0±0 |  | 0 | 0 | 0 | 0 | 0 | 0.0055 | 0.0059 | 0.0016286 ±0.0027837 | 0.1726229 |
| PAC001040_s | 0 | 0 | 0 | 0 | 0 | 0 | 0 | 0±0 |  | 0 | 0.006 | 0 | 0 | 0 | 0 | 0 | 0.0008571 ±0.0022678 | 0.3559177 |
| PAC001043_g_uc | 0 | 0 | 0 | 0 | 0 | 0 | 0 | 0±0 |  | 0 | 0 | 0 | 0.0026 | 0 | 0 | 0 | 0.0003714 ±0.0009827 | 0.3559177 |
| PAC001046_s | 0 | 0 | 0 | 0 | 0 | 0 | 0 | 0±0 |  | 0 | 0 | 0 | 0.0053 | 0 | 0 | 0 | 0.0007571 ±0.0020032 | 0.3559177 |
| PAC001048_s group | 0 | 0 | 0 | 0 | 0 | 0 | 0 | 0±0 |  | 0 | 0 | 0 | 0 | 0.0039 | 0 | 0 | 0.0005571 ±0.0014741 | 0.3559177 |
| PAC001079_s | 0 | 0 | 0 | 0 | 0 | 0.003 | 0.0033 | 0.0009 ±0.0015395 |  | 0 | 0 | 0 | 0 | 0.0077 | 0 | 0 | 0.0011 ±0.0029103 | 0.8887632 |
| PAC001095_s | 0 | 0 | 0 | 0 | 0 | 0.003 | 0 | 0.0004286 ±0.0011339 |  | 0 | 0 | 0 | 0 | 0.0077 | 0 | 0.003 | 0.0015286 ±0.0029421 | 0.4231569 |
| PAC001103_g_uc | 0 | 0 | 0 | 0 | 0 | 0 | 0.0067 | 0.0009571 ±0.0025324 |  | 0 | 0 | 0 | 0 | 0 | 0.0028 | 0 | 0.0004 ±0.0010583 | 0.62971 |
| PAC001104_s | 0 | 0 | 0 | 0 | 0.0031 | 0.0059 | 0 | 0.0012857 ±0.0023398 |  | 0 | 0 | 0 | 0 | 0 | 0.0055 | 0 | 0.0007857 ±0.0020788 | 0.2961204 |
| PAC001123_s | 0 | 0 | 0 | 0 | 0 | 0 | 0 | 0±0 |  | 0 | 0 | 0 | 0 | 0 | 0.0028 | 0 | 0.0004 ±0.0010583 | 0.3559177 |
| PAC001113_s | 0 | 0 | 0 | 0 | 0.0031 | 0 | 0 | 0.0004429 ±0.0011717 |  | 0 | 0 | 0 | 0 | 0 | 0 | 0 | 0±0 | 0.3559177 |
| PAC001119_s | 0 | 0 | 0 | 0 | 0.0031 | 0 | 0.0033 | 0.0009143 ±0.0015625 |  | 0 | 0 | 0 | 0 | 0 | 0 | 0 | 0±0 | 0.1725579 |
| PAC001121_s group | 0 | 0 | 0 | 0 | 0 | 0 | 0.0033 | 0.0004714 ±0.0012473 |  | 0 | 0 | 0 | 0 | 0 | 0 | 0 | 0±0 | 0.3559177 |
| PAC001125_s | 0 | 0 | 0 | 0 | 0 | 0 | 0.0033 | 0.0004714 ±0.0012473 |  | 0 | 0 | 0 | 0 | 0 | 0 | 0 | 0±0 | 0.3559177 |
| PAC001143_s | 0.0707 | 0.0536 | 0 | 0 | 0 | 0 | 0 | 0.0177571 ±0.0307252 |  | 0.1154 | 0.06 | 0 | 0 | 0 | 0 | 0 | 0.0250571 ±0.0456839 | 0.29047 |
| PAC001163_s | 0.2517 | 0.2997 | 0 | 0 | 10.4528 | 0.0059 | 1.6659 | 1.8108571 ±3.8564888 |  | 0.9782 | 0.3269 | 0.0026 | 0 | 5.1793 | 0.0055 | 3.4435 | 1.4194286 ±2.0673322 | 0.6617866 |
| PAC001165_s | 0 | 0 | 0 | 0 | 0.0031 | 0 | 0 | 0.0004429 ±0.0011717 |  | 0 | 0 | 0 | 0 | 0.0039 | 0 | 0 | 0.0005571 ±0.0014741 | 0.3559177 |
| PAC001173_s | 0 | 0 | 0.0025 | 0 | 0 | 0 | 0 | 0.0003571 ±0.0009449 |  | 0 | 0 | 0 | 0 | 0 | 0 | 0 | 0±0 | 0.3559177 |
| PAC001177_s | 0 | 0 | 0.0025 | 0 | 0 | 0.0059 | 0 | 0.0012 ±0.0022723 |  | 0 | 0 | 0 | 0 | 0 | 0 | 0 | 0±0 | 0.2118336 |
| PAC001178_s | 0 | 0 | 0 | 0 | 0.0815 | 0 | 2.7342 | 0.4022429 ±1.0287449 |  | 0 | 0 | 0 | 0 | 1.3151 | 0 | 0.0979 | 0.2018571 ±0.492248 | 0.6659497 |
| PAC001187_s | 0 | 0 | 0 | 0 | 0 | 0 | 0 | 0±0 |  | 0 | 0 | 0 | 0 | 0 | 0.0028 | 0 | 0.0004±0.0010583 | 0.3559177 |
| PAC001200_s | 0.0028 | 0 | 0 | 0 | 0.0063 | 0 | 0 | 0.0013 ±0.0024393 |  | 0.0027 | 0 | 0 | 0 | 0.0116 | 0 | 0.003 | 0.0024714 ±0.0042445 | 0.197822 |
| PAC001204_s | 0 | 0 | 0 | 0 | 0 | 0.003 | 0 | 0.0004286 ±0.0011339 |  | 0 | 0 | 0 | 0 | 0 | 0 | 0 | 0±0 | 0.3559177 |
| PAC001222_s | 0 | 0 | 0 | 0 | 0 | 0 | 0.0067 | 0.0009571 ±0.0025324 |  | 0 | 0 | 0 | 0 | 0 | 0 | 0 | 0±0 | 0.3559177 |
| PAC001224_s | 0.017 | 0 | 0 | 0 | 0 | 0 | 0 | 0.0024286 ±0.0064254 |  | 0.0357 | 0 | 0 | 0 | 0 | 0 | 0 | 0.0051 0.0134933 | 0.3559177 |
| PAC001229_s | 0 | 0 | 0 | 0.0024 | 0 | 0 | 0 | 0.0003429 ±0.0009071 |  | 0 | 0.009 | 0 | 0 | 0 | 0 | 0 | 0.0012857 ±0.0034017 | 0.5213412 |
| PAC001255_s | 0.789 | 0 | 0 | 0 | 0 | 0 | 0 | 0.1127143 ±0.298214 |  | 0.0137 | 0 | 0 | 0 | 0 | 0 | 0 | 0.0019571 ±0.0051781 | 0.3559177 |
| PAC001269_s | 0 | 0 | 0 | 0 | 0 | 0 | 0 | 0±0 |  | 0 | 0 | 0 | 0 | 0 | 0.0028 | 0 | 0.0004 ±0.0010583 | 0.3559177 |
| PAC001282_s | 0.6589 | 0.3375 | 0.0692 | 0.2679 | 0.0783 | 0 | 0.5141 | 0.2751286 ±0.246703 |  | 0.8903 | 0.2849 | 0.0732 | 6.6373 | 0.2198 | 0 | 0.0623 | 1.1668286 ±2.4308669 | 0.3681893 |
| PAC001292_s | 0 | 0 | 0 | 0 | 0 | 0 | 0 | 0±0 |  | 0 | 0 | 0 | 0 | 0 | 0 | 0.003 | 0.0004286 ±0.0011339 | 0.3559177 |
| PAC001294_s | 0 | 0 | 0 | 0 | 0 | 0 | 0.0033 | 0.0004714 ±0.0012473 |  | 0 | 0 | 0 | 0 | 0 | 0.0028 | 0.003 | 0.0008286 ±0.0014162 | 0.4164779 |
| PAC001304_s | 0 | 0 | 0 | 0.0047 | 0 | 0 | 0 | 0.0006714 ±0.0017764 |  | 0 | 0 | 0 | 0 | 0 | 0 | 0 | 0±0 | 0.3559177 |
| PAC001370_s | 0 | 0 | 0 | 0 | 0 | 0.003 | 0 | 0.0004286 ±0.0011339 |  | 0 | 0 | 0 | 0 | 0 | 0 | 0 | 0±0 | 0.3559177 |
| PAC001372_s | 0 | 0 | 0 | 0 | 0.0031 | 0 | 0 | 0.0004429 ±0.0011717 |  | 0 | 0 | 0 | 0 | 0 | 0 | 0 | 0±0 | 0.3559177 |
| PAC001304_s | 0 | 0 | 0 | 0 | 0 | 0 | 0 | 0±0 |  | 0 | 0.003 | 0 | 0 | 0 | 0 | 0 | 0.0004286 ±0.0011339 | 0.3559177 |
| PAC001319_s | 0 | 0 | 0 | 0 | 0 | 0 | 0 | 0±0 |  | 0 | 0 | 0 | 0 | 0 | 0 | 0.0059 | 0.0008429 ±0.00223 | 0.3559177 |
| PAC001379_s | 0 | 0 | 0 | 0 | 0 | 0 | 0 | 0±0 |  | 0 | 0 | 0 | 0 | 0 | 0.0028 | 0 | 0.0004 ±0.0010583 | 0.3559177 |
| PAC001450_s | 0.8031 | 1.7696 | 0 | 0.0024 | 11.6121 | 0.0089 | 0.0134 | 2.0299286 ±4.2773301 |  | 2.1021 | 6.6451 | 0 | 0 | 21.5233 | 0.0055 | 0.0089 | 4.3264143 ±7.9666357 | 0.1612235 |
| PAC001454_s | 0.0028 | 0 | 0 | 0 | 0 | 0 | 0 | 0.0004 ±0.0010583 |  | 0 | 0 | 0 | 0 | 0 | 0 | 0 | 0±0 | 0.3559177 |
| PAC001465_s | 0 | 0.0032 | 0 | 0 | 0 | 0 | 0 | 0.0004571 ±0.0012095 |  | 0 | 0 | 0 | 0 | 0 | 0 | 0 | 0±0 | 0.3559177 |
| PAC001488_s group | 0 | 0 | 0 | 0 | 0 | 0.003 | 0 | 0.0004286 ±0.0011339 |  | 0 | 0 | 0 | 0 | 0 | 0 | 0 | 0±0 | 0.3559177 |
| PAC001500_s | 0 | 0 | 0 | 0 | 0 | 0 | 0.0033 | 0.0004714 ±0.0012473 |  | 0 | 0 | 0 | 0 | 0 | 0 | 0 | 0±0 | 0.3559177 |
| PAC001515_s | 0 | 0 | 0 | 0 | 0 | 0 | 0.0033 | 0.0004714 ±0.0012473 |  | 0 | 0 | 0 | 0 | 0 | 0 | 0 | 0±0 | 0.3559177 |
| PAC001473_s | 0 | 0 | 0 | 0 | 0 | 0 | 0 | 0±0 |  | 0 | 0 | 0 | 0 | 0.0039 | 0 | 0 | 0.0005571±0.0014741 | 0.3559177 |
| PAC001548_s | 0 | 0 | 0 | 0 | 0 | 0 | 0 | 0±0 |  | 0 | 0 | 0 | 0 | 0 | 0 | 0.003 | 0.0004286 ±0.0011339 | 0.3559177 |
| PAC001557_s | 0 | 0 | 0 | 0 | 0 | 0 | 0 | 0±0 |  | 0 | 0 | 0 | 0 | 0 | 0 | 0.003 | 0.0004286 ±0.0011339 | 0.3559177 |
| PAC001574_s group | 0 | 0 | 0 | 0 | 0.0031 | 0 | 0 | 0.0004429 ±0.0011717 |  | 0 | 0 | 0 | 0 | 0 | 0.0028 | 0 | 0.0004 ±0.0010583 | 0.9491299 |
| PAC001585_s group | 0 | 0 | 0 | 0 | 0.0031 | 0.003 | 0 | 0.0008714 ±0.0014885 |  | 0 | 0 | 0 | 0 | 0 | 0 | 0 | 0±0 | 0.172377 |
| PAC001587_s | 0 | 0 | 0 | 0 | 0 | 0 | 0 | 0±0 |  | 0 | 0 | 0 | 0 | 0 | 0.0028 | 0 | 0.0004 ±0.0010583 | 0.3559177 |
| PAC001597_s | 0 | 0 | 0.0025 | 0.0213 | 0 | 0 | 0 | 0.0034 ±0.007948 |  | 0 | 0 | 0 | 0.2087 | 0 | 0 | 0 | 0.0298143 ±0.0788812 | 0.3629405 |
| PAC001601_s | 0 | 0 | 0 | 0 | 0.0031 | 0 | 0 | 0.0004429 ±0.0011717 |  | 0 | 0 | 0 | 0 | 0 | 0 | 0 | 0±0 | 0.3559177 |
| PAC001654_s | 0 | 0 | 0 | 0 | 0 | 0 | 0 | 0±0 |  | 0 | 0 | 0 | 0 | 0 | 0.0028 | 0 | 0.0004 0.0010583 | 0.3559177 |
| PAC001666_s | 0 | 0 | 0 | 0 | 0 | 0 | 0 | 0±0 |  | 0 | 0 | 0 | 0 | 0 | 0 | 0.003 | 0.0004286 ±0.0011339 | 0.3559177 |
| PAC001671_s | 0.0509 | 0.0063 | 0 | 0 | 0 | 0 | 0 | 0.0081714 ±0.0189873 |  | 0.0055 | 0 | 0 | 0 | 0 | 0 | 0 | 0.0007857 ±0.0020788 | 0.2922128 |
| PAC001695_s | 0 | 0 | 0 | 0 | 0 | 0 | 0.0033 | 0.0004714 ±0.0012473 |  | 0 | 0 | 0 | 0 | 0 | 0 | 0 | 0±0 | 0.3559177 |
| PAC001679_s | 0 | 0 | 0 | 0 | 0 | 0 | 0 | 0±0 |  | 0 | 0 | 0 | 0 | 0 | 0 | 0.003 | 0.0004286 ±0.0011339 | 0.3559177 |
| PAC001710_s | 0 | 0 | 0 | 0 | 0.0031 | 0 | 0 | 0.0004429 ±0.0011717 |  | 0 | 0 | 0 | 0 | 0 | 0.0028 | 0 | 0.0004 0.0010583 | 0.9491299 |
| PAC001711_s | 0 | 0 | 0 | 0 | 0 | 0 | 0 | 0±0 |  | 0 | 0.003 | 0 | 0 | 0 | 0 | 0 | 0.0004286 ±0.0011339 | 0.3559177 |
| PAC001726_s | 0 | 0 | 0 | 0 | 0 | 0 | 0 | 0±0 |  | 0 | 0 | 0 | 0 | 0 | 0 | 0.003 | 0.0004286 ±0.0011339 | 0.3559177 |
| PAC001727_s group | 0 | 0 | 0 | 0 | 0 | 0 | 0 | 0±0 |  | 0 | 0.006 | 0 | 0.0079 | 0.0116 | 0.0139 | 0.0059 | 0.0064714 ±0.0052889 | 0.0177467 |
| PAC001741_s | 0 | 0 | 0 | 0 | 0 | 0 | 0 | 0±0 |  | 0 | 0 | 0 | 0 | 0 | 0.0028 | 0 | 0.0004 ±0.0010583 | 0.3559177 |
| PAC001728_s group | 0 | 0 | 0 | 0 | 0.0031 | 0 | 0 | 0.0004429 ±0.0011717 |  | 0 | 0 | 0 | 0 | 0 | 0 | 0 | 0±0 | 0.3559177 |
| PAC001731_s | 0 | 0 | 0 | 0 | 0 | 0 | 0.0033 | 0.0004714 ±0.0012473 |  | 0 | 0 | 0 | 0 | 0 | 0 | 0 | 0±0 | 0.3559177 |
| PAC001740_s | 0 | 0 | 0 | 0 | 0.0031 | 0 | 0 | 0.0004429 ±0.0011717 |  | 0 | 0 | 0 | 0 | 0 | 0 | 0 | 0±0 | 0.3559177 |
| PAC001744_s group | 0 | 0 | 0 | 0 | 0 | 0.003 | 0 | 0.0004286 ±0.0011339 |  | 0 | 0 | 0 | 0 | 0 | 0 | 0 | 0±0 | 0.3559177 |
| PAC001757_s group | 0 | 0 | 0 | 0 | 0 | 0.003 | 0.0033 | 0.0009 ±0.0015395 |  | 0 | 0 | 0 | 0 | 0 | 0 | 0.003 | 0.0004286 ±0.0011339 | 0.3082762 |
| PAC001902_s | 0 | 0 | 0 | 0 | 0 | 0 | 0.0033 | 0.0004714 ±0.0012473 |  | 0 | 0 | 0 | 0 | 0 | 0 | 0 | 0±0 | 0.3559177 |
| PAC001782_s | 0 | 0 | 0 | 0 | 0 | 0 | 0 | 0±0 |  | 0 | 0 | 0 | 0 | 0 | 0 | 0.003 | 0.0004286 ±0.0011339 | 0.3559177 |
| PAC001784_s | 0 | 0 | 0 | 0 | 0 | 0 | 0 | 0±0 |  | 0 | 0 | 0 | 0 | 0 | 0.0028 | 0 | 0.0004 0.0010583 | 0.3559177 |
| PAC001901_s | 0 | 0 | 0 | 0 | 0 | 0 | 0 | 0±0 |  | 0 | 0 | 0 | 0 | 0 | 0 | 0.003 | 0.0004286 ±0.0011339 | 0.3559177 |
| PAC001981_s group | 0 | 0 | 0 | 0 | 0.0063 | 0.003 | 0.0033 | 0.0018 ±0.0024799 |  | 0 | 0 | 0 | 0 | 0.0077 | 0.0139 | 0.003 | 0.0035143 ±0.0054014 | 0.3097385 |
| PAC002009_s group | 0 | 0 | 0 | 0 | 0 | 0.003 | 0.0033 | 0.0009 ±0.0015395 |  | 0 | 0 | 0 | 0 | 0 | 0 | 0.0059 | 0.0008429 ±0.00223 | 0.9286631 |
| PAC002315_s | 0.3789 | 0.0095 | 0.1285 | 0.6757 | 0.3603 | 0 | 0.4707 | 0.2890857 ±0.2526918 |  | 0.1017 | 0.081 | 0.306 | 0.5811 | 0.0848 | 0 | 0.3025 | 0.2081571 ±0.201498 | 0.263472 |
| PAC002344_s | 0 | 0 | 0 | 0 | 0 | 0.003 | 0 | 0.0004286 ±0.0011339 |  | 0 | 0 | 0 | 0 | 0 | 0 | 0 | 0±0 | 0.3559177 |
| PAC002364_s | 0.1159 | 0.0032 | 0.0049 | 0.0024 | 0 | 0 | 0 | 0.0180571 ±0.0431864 |  | 0.011 | 0 | 0 | 0 | 0 | 0 | 0 | 0.0015714 ±0.0041576 | 0.3065453 |
| PAC002401_s group | 0 | 0 | 0 | 0 | 0 | 0 | 0.0033 | 0.0004714 ±0.0012473 |  | 0 | 0 | 0 | 0 | 0 | 0 | 0 | 0±0 | 0.3559177 |
| PAC002403_s | 0 | 0 | 0 | 0 | 0 | 0 | 0 | 0±0 |  | 0 | 0 | 0 | 0 | 0 | 0.0028 | 0 | 0.0004 ±0.0010583 | 0.3559177 |
| PAC002442_s | 0 | 0 | 0 | 0 | 0 | 0 | 0.0033 | 0.0004714 ±0.0012473 |  | 0 | 0 | 0 | 0 | 0 | 0.0028 | 0 | 0.0004 ±0.0010583 | 0.9182286 |
| PAC002512_s group | 0 | 0 | 0 | 0 | 0.0031 | 0 | 0 | 0.0004429 ±0.0011717 |  | 0 | 0 | 0 | 0 | 0 | 0 | 0 | 0±0 | 0.3559177 |
| PAC002523_s | 0 | 0 | 0 | 0 | 0 | 0.003 | 0 | 0.0004286 ±0.0011339 |  | 0 | 0 | 0 | 0 | 0 | 0 | 0 | 0±0 | 0.3559177 |
| PAC002522_s | 0 | 0 | 0 | 0 | 0 | 0 | 0 | 0±0 |  | 0 | 0 | 0 | 0 | 0.0039 | 0 | 0 | 0.0005571 ±0.0014741 | 0.3559177 |
| Paenibacillus polymyxa group | 0 | 0 | 0 | 0 | 0 | 0 | 0 | 0±0 |  | 0 | 0 | 0 | 0.0053 | 0 | 0 | 0 | 0.0007571 ±0.0020032 | 0.3559177 |
| Paenibacillus cookii group | 0 | 0 | 0 | 0 | 0.0031 | 0 | 0 | 0.0004429 ±0.0011717 |  | 0 | 0 | 0 | 0 | 0 | 0 | 0 | 0±0 | 0.3559177 |
| Paenibacillus ginsengihumi | 0 | 0 | 0 | 0 | 0.0063 | 0 | 0 | 0.0009 ±0.0023812 |  | 0 | 0 | 0 | 0 | 0 | 0 | 0 | 0±0 | 0.3559177 |
| Paenibacillus lentus group | 0 | 0 | 0 | 0 | 0.0031 | 0 | 0 | 0.0004429 ±0.0011717 |  | 0 | 0 | 0 | 0 | 0 | 0 | 0 | 0±0 | 0.3559177 |
| Paenibacillus woosongensis | 0 | 0 | 0 | 0 | 0.0031 | 0 | 0 | 0.0004429 ±0.0011717 |  | 0 | 0 | 0 | 0 | 0 | 0 | 0 | 0±0 | 0.3559177 |
| Paeniclostridium ghonii group | 0 | 0.0095 | 0.0049 | 0 | 2.1965 | 0.003 | 0 | 0.3162714 ±0.8291102 |  | 0.0082 | 0 | 0 | 0.0317 | 0 | 0 | 0.2343 | 0.0391714 0.0868215 | 0.4219502 |
| Parabacteroides merdae | 0 | 0 | 0 | 0 | 0 | 0.003 | 0 | 0.0004286 ±0.0011339 |  | 0 | 0.003 | 0 | 0 | 0 | 0 | 0 | 0.0004286 ±0.0011339 | 1 |
| Peptostreptococcus canis | 0 | 0 | 0 | 0 | 0 | 0 | 0 | 0±0 |  | 0 | 0.009 | 0 | 0.0211 | 0 | 0 | 0 | 0.0043 ±0.008132 | 0.2113257 |
| Prevotella melaninogenica | 0 | 0 | 0 | 0 | 0 | 0 | 0 | 0±0 |  | 0 | 0.009 | 0 | 0 | 0 | 0 | 0 | 0.0012857 ±0.0034017 | 0.3559177 |
| Prevotella salivae | 0 | 0 | 0 | 0 | 0 | 0 | 0 | 0±0 |  | 0 | 0.003 | 0 | 0 | 0 | 0 | 0 | 0.0004286±0.0011339 | 0.3559177 |
| Paracoccus denitrificans group | 0 | 0 | 0 | 0 | 0 | 0.003 | 0 | 0.0004286 ±0.0011339 |  | 0 | 0 | 0 | 0 | 0 | 0 | 0 | 0±0 | 0.3559177 |
| Parasutterella excrementihominis group | 0 | 0 | 0.0025 | 0 | 0 | 0 | 0 | 0.0003571 ±0.0009449 |  | 0 | 0 | 0 | 0 | 0 | 0 | 0 | 0±0 | 0.3559177 |
| Parvibacter caecicola | 0 | 0 | 0 | 0 | 0 | 0.003 | 0.0033 | 0.0009 ±0.0015395 |  | 0 | 0 | 0 | 0 | 0 | 0 | 0 | 0±0 | 0.1728876 |
| Pediococcus acidilactici group | 0 | 0 | 0 | 0 | 0.0094 | 0 | 0 | 0.0013429 ±0.0035529 |  | 0 | 0 | 0 | 0 | 0 | 0 | 0 | 0±0 | 0.3559177 |
| Porphyromonas cangingivalis | 0.0028 | 0 | 0 | 0 | 0 | 0 | 0 | 0.0004 ±0.0010583 |  | 0 | 0 | 0 | 0 | 0 | 0 | 0 | 0±0 | 0.3559177 |
| Prevotella_uc | 0 | 0 | 0 | 0.0047 | 0 | 0 | 0 | 0.0006714 ±0.0017764 |  | 0 | 0 | 0 | 0 | 0 | 0 | 0 | 0±0 | 0.3559177 |
| Proteus mirabilis | 0 | 0.041 | 0 | 0 | 0 | 0 | 0 | 0.0058571 ±0.0154965 |  | 0 | 0.003 | 0 | 0 | 0 | 0 | 0 | 0.0004286 ±0.0011339 | 0.3559177 |
| Pseudomonas fulva group | 0 | 0 | 0 | 0 | 0 | 0 | 0 | 0±0 |  | 0 | 0 | 0 | 0.0026 | 0 | 0 | 0 | 0.0003714 ±0.0009827 | 0.3559177 |
| Robinsoniella peoriensis | 0.0028 | 0 | 0 | 0 | 0 | 0 | 0 | 0.0004 ±0.0010583 |  | 0.011 | 0 | 0 | 0 | 0 | 0 | 0 | 0.0015714 ±0.0041576 | 0.3559177 |
| Robinsoniella_uc | 0.0283 | 0 | 0 | 0 | 0 | 0 | 0 | 0.0040429 ±0.0106964 |  | 0.1676 | 0 | 0 | 0 | 0 | 0 | 0 | 0.0239429 ±0.0633468 | 0.3559177 |
| Romboutsia sedimentorum | 0.0339 | 1.6592 | 0.0124 | 0 | 0.1065 | 0 | 3.9193 | 0.8187571 ±1.4963266 |  | 0.1456 | 0.009 | 0 | 0.0132 | 0.0424 | 0 | 2.7732 | 0.4262 ±1.0362083 | 0.1906605 |
| Romboutsia timonensis | 0.0028 | 0.0126 | 0 | 0 | 0.0219 | 0.0118 | 0.03 | 0.0113 ±0.0114864 |  | 0.0055 | 0.006 | 0 | 0 | 0.0116 | 0.1746 | 0.0297 | 0.0324857 ±0.0634833 | 0.4051206 |
| Romboutsia_uc | 0 | 0.0032 | 0 | 0 | 0 | 0 | 0.01 | 0.0018857 ±0.0037716 |  | 0 | 0 | 0 | 0 | 0 | 0 | 0.003 | 0.0004286 ±0.0011339 | 0.2060922 |
| Roseburia cecicola group | 0 | 0 | 0 | 0 | 0 | 0 | 0 | 0±0 |  | 0 | 0 | 0.0026 | 0 | 0.0077 | 0 | 0 | 0.0014714 ±0.0029125 | 0.2297768 |
| Roseburia intestinalis group | 0.0707 | 0.0852 | 0.0148 | 0 | 0 | 0 | 0.0267 | 0.0282 ±0.0356436 |  | 0 | 0.009 | 0.0183 | 0 | 0.0231 | 0 | 0 | 0.0072 ±0.0098879 | 0.2009015 |
| Rothia nasimurium | 0 | 0 | 0 | 0 | 0 | 0.003 | 0 | 0.0004286 ±0.0011339 |  | 0 | 0 | 0 | 0 | 0 | 0 | 0 | 0±0 | 0.3559177 |
| Ruminococcus bromii | 0 | 0 | 0 | 0 | 0 | 0 | 0 | 0±0 |  | 0 | 0 | 0 | 0.0053 | 0 | 0 | 0 | 0.0007571 ±0.0020032 | 0.3559177 |
| Ruminococcus gnavus | 1.9202 | 3.5487 | 2.9232 | 12.1651 | 20.7677 | 1.0829 | 3.0947 | 6.5003571 ±7.2924607 |  | 8.7272 | 9.6018 | 2.4424 | 17.268 | 17.6668 | 4.5174 | 43.6766 | 14.842886 ±13.967206 | 0.1830034 |
| Ruminococcus lactaris | 0 | 0 | 0.0025 | 0 | 0 | 0 | 0 | 0.0003571 ±0.0009449 |  | 0 | 0 | 0 | 0 | 0 | 0 | 0 | 0±0 | 0.3559177 |
| Ruminococcus torques | 0 | 0 | 0 | 0 | 0.0031 | 0 | 0 | 0.0004429 ±0.0011717 |  | 0 | 0 | 0 | 0 | 0 | 0 | 0 | 0±0 | 0.3559177 |
| Ruminococcus torques | 0 | 0 | 0 | 0 | 0 | 0 | 0 | 0±0 |  | 0 | 0 | 0 | 0.0026 | 0 | 0 | 0 | 0.0003714 ±0.0009827 | 0.3559177 |
| Ruminococcus_g4_uc | 0 | 0 | 0 | 0 | 0 | 0 | 0 | 0±0 |  | 0.0027 | 0 | 0 | 0 | 0 | 0 | 0 | 0.0003857 ±0.0010205 | 0.3559177 |
| Saccharomonospora viridis | 0 | 0 | 0 | 0 | 0.0031 | 0 | 0 | 0.0004429 ±0.0011717 |  | 0 | 0 | 0 | 0 | 0 | 0 | 0 | 0±0 | 0.3559177 |
| Saccharimonas_uc | 0 | 0 | 0 | 0 | 0 | 0 | 0 | 0±0 |  | 0 | 0 | 0 | 0.0026 | 0.0039 | 0 | 0 | 0.0009286 ±0.0016296 | 0.1823923 |
| Sporichthya polymorpha group | 0 | 0 | 0 | 0 | 0 | 0 | 0 | 0±0 |  | 0 | 0.003 | 0 | 0 | 0 | 0 | 0 | 0.0004286 0.0011339 | 0.3559177 |
| Staphylococcus aureus group | 0 | 0 | 0 | 0 | 0.0188 | 0 | 0.0033 | 0.0031571 ±0.0070066 |  | 0 | 0 | 0 | 0 | 0.0193 | 0 | 0 | 0.0027571 0.0072947 | 0.4441247 |
| Staphylococcus carnosus group | 0 | 0 | 0 | 0 | 0 | 0 | 0 | 0±0 |  | 0 | 0 | 0 | 0 | 0.0077 | 0 | 0 | 0.0011 ±0.0029103 | 0.3559177 |
| Staphylococcus saprophyticus group | 0 | 0 | 0 | 0 | 0.0031 | 0 | 0 | 0.0004429 ±0.0011717 |  | 0 | 0.006 | 0 | 0 | 0 | 0 | 0 | 0.0008571 ±0.0022678 | 0.700989 |
| Staphylococcus schleiferi group | 0 | 0 | 0 | 0 | 0 | 0 | 0 | 0±0 |  | 0.0055 | 0 | 0 | 0 | 0.0039 | 0 | 0 | 0.0013429 ±0.0023394 | 0.1796407 |
| Streptococcus canis | 0 | 0 | 0 | 0 | 0 | 0 | 0 | 0±0 |  | 0 | 0 | 0 | 0.177 | 0 | 0 | 0 | 0.0252857 ±0.0668997 | 0.3559177 |
| Staphylococcus simulans | 0 | 0.0032 | 0 | 0 | 0 | 0 | 0 | 0.0004571 ±0.0012095 |  | 0 | 0 | 0 | 0 | 0 | 0 | 0 | 0±0 | 0.3559177 |
| Streptococcus anginosus group | 0 | 0 | 0 | 0 | 0.0125 | 0 | 0 | 0.0017857 ±0.0047246 |  | 0 | 0 | 0 | 0 | 0 | 0 | 0 | 0±0 | 0.3559177 |
| Streptococcus fryi | 0 | 0 | 0 | 0.0071 | 0 | 0 | 0 | 0.0010143 ±0.0026835 |  | 0.0027 | 0.003 | 0 | 0.0211 | 0 | 0 | 0 | 0.0038286 ±0.0077341 | 0.1955623 |
| Streptococcus minor | 0 | 0 | 0 | 0 | 0 | 0 | 0 | 0±0 |  | 0 | 0 | 0 | 0.0026 | 0 | 0 | 0 | 0.0003714 ±0.0009827 | 0.3559177 |
| Streptococcus gallolyticus group | 0 | 0 | 0 | 0 | 0 | 0.4013 | 0 | 0.0573286 ±0.1516771 |  | 0 | 0 | 0 | 0 | 0 | 0 | 0 | 0±0 | 0.3559177 |
| Streptococcus salivarius group | 0 | 0.0032 | 0 | 0 | 1.0246 | 0 | 0 | 0.1468286 ±0.3870627 |  | 0.0027 | 0 | 0 | 0.0026 | 0 | 0 | 0.003 | 0.0011857 ±0.0014837 | 0.3585219 |
| Streptococcus sinensis group | 0 | 0 | 0 | 0 | 0 | 0 | 0 | 0±0 |  | 0 | 0.003 | 0 | 0 | 0 | 0 | 0 | 0.0004286 ±0.0011339 | 0.3559177 |
| Sulfurovum aggregans | 0 | 0 | 0 | 0 | 0 | 0.0059 | 0 | 0.0008429 ±0.00223 |  | 0 | 0 | 0 | 0 | 0.0077 | 0 | 0 | 0.0011 ±0.0029103 | 0.8689293 |
| Sutterella wadsworthensis | 0 | 0 | 0 | 0 | 0 | 0 | 0 | 0±0 |  | 0 | 0 | 0.0026 | 0 | 0 | 0 | 0 | 0.0003714 ±0.0009827 | 0.3559177 |
| Sutterella_uc | 0.0028 | 0 | 0 | 0 | 0 | 0 | 0 | 0.0004 ±0.0010583 |  | 0.0027 | 0.006 | 0 | 0 | 0 | 0 | 0 | 0.0012429 ±0.0023266 | 0.3647154 |
| Terrisporobacter glycolicus group | 0 | 0.0095 | 0 | 0 | 0 | 0 | 0.0534 | 0.0089857 ±0.0199023 |  | 0 | 0 | 0 | 0 | 0.0039 | 0.0111 | 0.0119 | 0.0038429 ±0.0054261 | 0.4578928 |
| Terrisporobacter petrolearius | 0.0057 | 0.6119 | 0 | 0 | 0.3102 | 0 | 6.8104 | 1.1054571 ±2.5264592 |  | 0 | 0 | 0 | 0 | 0 | 0 | 0 | 0±0 | 0.2910027 |
| Terrisporobacter petrolearius | 0 | 0 | 0 | 0 | 0 | 0 | 0 | 0±0 |  | 0.0055 | 0.039 | 0 | 0.0106 | 0.0424 | 0.643 | 1.6669 | 0.3439143 ±0.6281622 | 0.1976359 |
| Terrisporobacter_uc | 0 | 0 | 0 | 0 | 0.0031 | 0 | 0.03 | 0.0047286 ±0.0112034 |  | 0 | 0 | 0 | 0 | 0 | 0 | 0.003 | 0.0004286 ±0.0011339 | 0.3019791 |
| Treponema porcinum | 0 | 0 | 0 | 0 | 0 | 0 | 0 | 0±0 |  | 0 | 0 | 0 | 0 | 0 | 0.0028 | 0 | 0.0004 ±0.0010583 | 0.3559177 |
| Thermoactinomyces vulgaris | 0 | 0 | 0 | 0 | 0.0063 | 0 | 0 | 0.0009 ±0.0023812 |  | 0 | 0 | 0 | 0 | 0 | 0 | 0 | 0±0 | 0.3559177 |
| Tissierella praeacuta | 0 | 0 | 0 | 0.0024 | 0 | 0 | 0 | 0.0003429 ±0.0009071 |  | 0 | 0 | 0 | 0 | 0 | 0 | 0 | 0±0 | 0.3559177 |
| Turicibacter sanguinis | 0.0057 | 0.4164 | 0.042 | 0 | 0.9839 | 0.0561 | 0 | 0.2148714 ±0.370406 |  | 1.9345 | 0 | 0.0183 | 0.0211 | 0 | 0.2439 | 2.1918 | 0.6299429 ±0.9856258 | 0.3916756 |
| Ureibacillus thermosphaericus | 0 | 0 | 0 | 0 | 0.0094 | 0 | 0 | 0.0013429 ±0.0035529 |  | 0 | 0 | 0 | 0 | 0.0039 | 0 | 0 | 0.0005571 ±0.0014741 | 0.3559177 |
| Veillonella dispar | 0 | 0.0032 | 0 | 0 | 0.0031 | 0 | 0 | 0.0009 ±0.0015373 |  | 0 | 0.006 | 0 | 0 | 0 | 0 | 0 | 0.0008571 ±0.0022678 | 0.9491299 |
| Veillonella parvula group | 0 | 0 | 0 | 0 | 0 | 0 | 0 | 0±0 |  | 0.0027 | 0 | 0 | 0 | 0 | 0 | 0 | 0.0003857 ±0.0010205 | 0.3559177 |
| Weissella confusa group | 0 | 0 | 0 | 0 | 0 | 0 | 0.0067 | 0.0009571 ±0.0025324 |  | 0 | 0 | 0 | 0 | 0 | 0 | 0 | 0±0 | 0.3559177 |
| Unclassified in higher taxonomic rank | 0.017 | 0.0095 | 0.0025 | 0.0024 | 0.0376 | 0.0148 | 0.01 | 0.0134 ±0.0120218 |  | 0.0082 | 0.03 | 0 | 0.0343 | 0.0386 | 0.0416 | 0.0119 | 0.0235143 ±0.0165096 | 0.1446159 |

Diversity index (Shannon)* : Quantitative indicator of the number of different bacteria that are present in the sample. (Magurran 2013)

Diversity index (Jackknife)** : Indicator of species richness that is sensitive to rare and abundant OTUs (Operational Taxonomic Unit). Higher values indicate higher diversity. (Burnham et al. 1979)

Diversity index (Simpson)*** : Indicator of species evenness that displays the probability that two randomly selected sequences are of the same species. Values range from 0 to 1, and lower values indicate higher diversity. (Magurran 2013)

Good's coverage (%) **** : Indicator of the extent to which the number of sequencing reads used for analysis represents the actual species population of the sample. The value can range from 0 to 100%, with 100% indicating a complete sampling of species, meaning that additional sequencing is unlikely to find any more new species. (Good 1953)

† Bacterial species used in Figure 2 are highlighted in yellow.

**References**

Magurran, A. E. (2013). Measuring biological diversity. John Wiley & Sons.

Burnham, K. P. & Overton, W. S. (1979) Robust estimation of population size when capture probabilities vary among animals. Ecology, 60, 927-936.

Good, I. J. (1953) The population frequencies of species and the estimation of population parameters. Biometrika, 237-264.
